# Boronate-Based Oxidant-Responsive Derivatives of Acetaminophen as Proinhibitors of Myeloperoxidase

**DOI:** 10.1021/acs.chemrestox.3c00140

**Published:** 2023-08-03

**Authors:** Karolina Pierzchała, Jakub Pięta, Marlena Pięta, Monika Rola, Jacek Zielonka, Adam Sikora, Andrzej Marcinek, Radosław Michalski

**Affiliations:** † Institute of Applied Radiation Chemistry, Department of Chemistry, Lodz University of Technology, Zeromskiego 116, 90-924 Lodz, Poland; ‡ Department of Biophysics and Free Radical Research Center, Medical College of Wisconsin, 8701 Watertown Plank Road, Milwaukee, Wisconsin 53226, United States

## Abstract

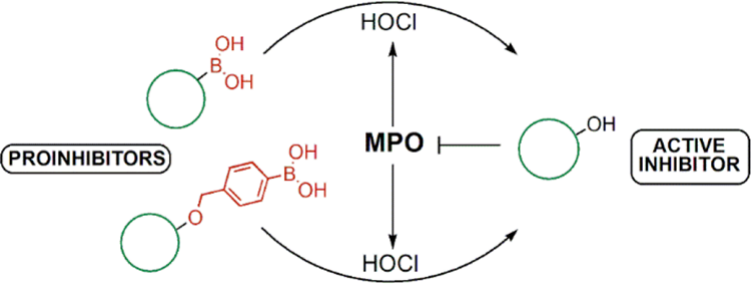

Myeloperoxidase (MPO) is an important component of the human innate immune system and the main source of a strong oxidizing and chlorinating species, hypochlorous acid (HOCl). Inadvertent, misplaced, or excessive generation of HOCl by MPO is associated with multiple human inflammatory diseases. Therefore, there is a considerable interest in the development of MPO inhibitors. Here, we report the synthesis and characterization of a boronobenzyl derivative of acetaminophen (AMBB), which can function as a proinhibitor of MPO and release acetaminophen, the inhibitor of chlorination cycle of MPO, in the presence of inflammatory oxidants, i.e., hydrogen peroxide, hypochlorous acid, or peroxynitrite. We demonstrate that the AMBB proinhibitor undergoes conversion to acetaminophen by all three oxidants, with the involvement of the primary phenolic product intermediate, with relatively long half-life at pH 7.4. The determined rate constants of the reaction of the AMBB proinhibitor with hydrogen peroxide, hypochlorous acid, or peroxynitrite are equal to 1.67, 1.6 × 10^4^, and 1.0 × 10^6^ M^–1^ s^–1^, respectively. AMBB showed lower MPO inhibitory activity (IC_50_ > 0.3 mM) than acetaminophen (IC_50_ = 0.14 mM) toward MPO-dependent HOCl generation. Finally, based on the determined reaction kinetics and the observed inhibitory effects of two plasma components, uric acid and albumin, on the extent of AMBB oxidation by ONOO^–^ and HOCl, we conclude that ONOO^–^ is the most likely potential activator of AMBB in human plasma.

## Introduction

**Scheme 1:**
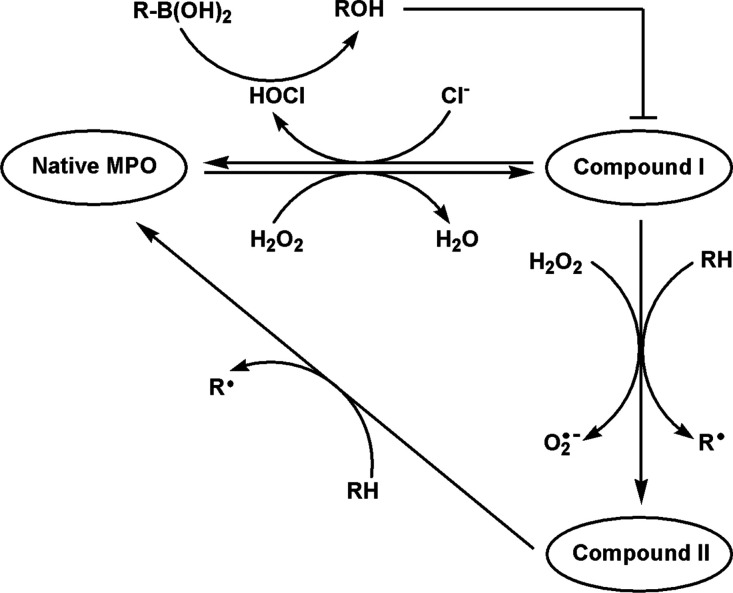
Redox Cycle of MPO

Myeloperoxidase (MPO) belongs to the *heme*-peroxidases family of enzymes and is a crucial component of the host’s immune defense system. It is present in neutrophils and monocytes circulating in blood and in tissue macrophages. Depending on the substrate availability, this enzyme may produce different one- and two-electron oxidants, but its microbicidal activity is mostly attributed to the production of hypochlorous acid (HOCl) from the chloride anion and hydrogen peroxide (H_2_O_2_), the major co-substrates for the enzyme at their physiological concentrations (Scheme 1).^1−5^

**Scheme 2:**
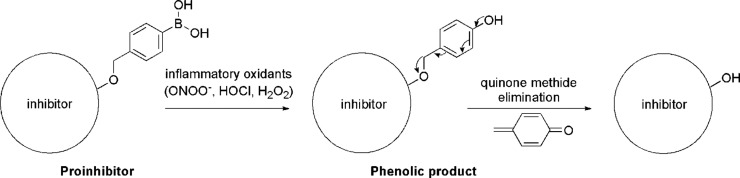
Oxidative Conversion of the MPO Chlorination Cycle Proinhibitor to the Active Form

However, sustained production of MPO-derived oxidants generated by MPO released to the extracellular matrix contributes to the initiation and propagation of inflammatory diseases.^1,2,5−8^ Thus, development of inhibitors that can modulate MPO activity at the site of inflammation to minimize oxidative damage to the host cells is of considerable interest. Nonetheless, many of the potential inhibitors of MPO (e.g., hydrazides) are inherently toxic due to their interference with other biological targets.^4^ One of the strategies that can enable site-specific inhibition of MPO is the use of oxidant-activated proinhibitors, the compounds that release the active form of inhibitors only in the presence of HOCl or other oxidants typically produced at sites of inflammation like H_2_O_2_ and peroxynitrite (ONOO^–^). The inhibitors substituted with the arylboronic acid moiety match to the proposed approach. Boronic acids react with HOCl, ONOO^–^, and H_2_O_2_ directly and with well-established stoichiometry forming corresponding phenols.^9,10^ Boronate-substituted bioactive agents have previously been developed as H_2_O_2_- or ONOO^–^-activated prodrugs.^11−13^ Here, we designed a boronate-based MPO proinhibitor by boronobenzylation of the bioactive phenolic drug. We envisioned that oxidation of the arylboronic acid moiety of the proinhibitor would lead to the formation of an unstable phenolic derivative, which subsequently would release an active inhibitor via quinone methide (QM) elimination (Scheme 2). For our study, we have selected an FDA-approved over-the-counter drug, acetaminophen (AM), which inhibits MPO-mediated HOCl production and possesses a hydroxyl group that can be conveniently substituted with a boronobenzyl moiety.^14,15^

**Scheme 3:**
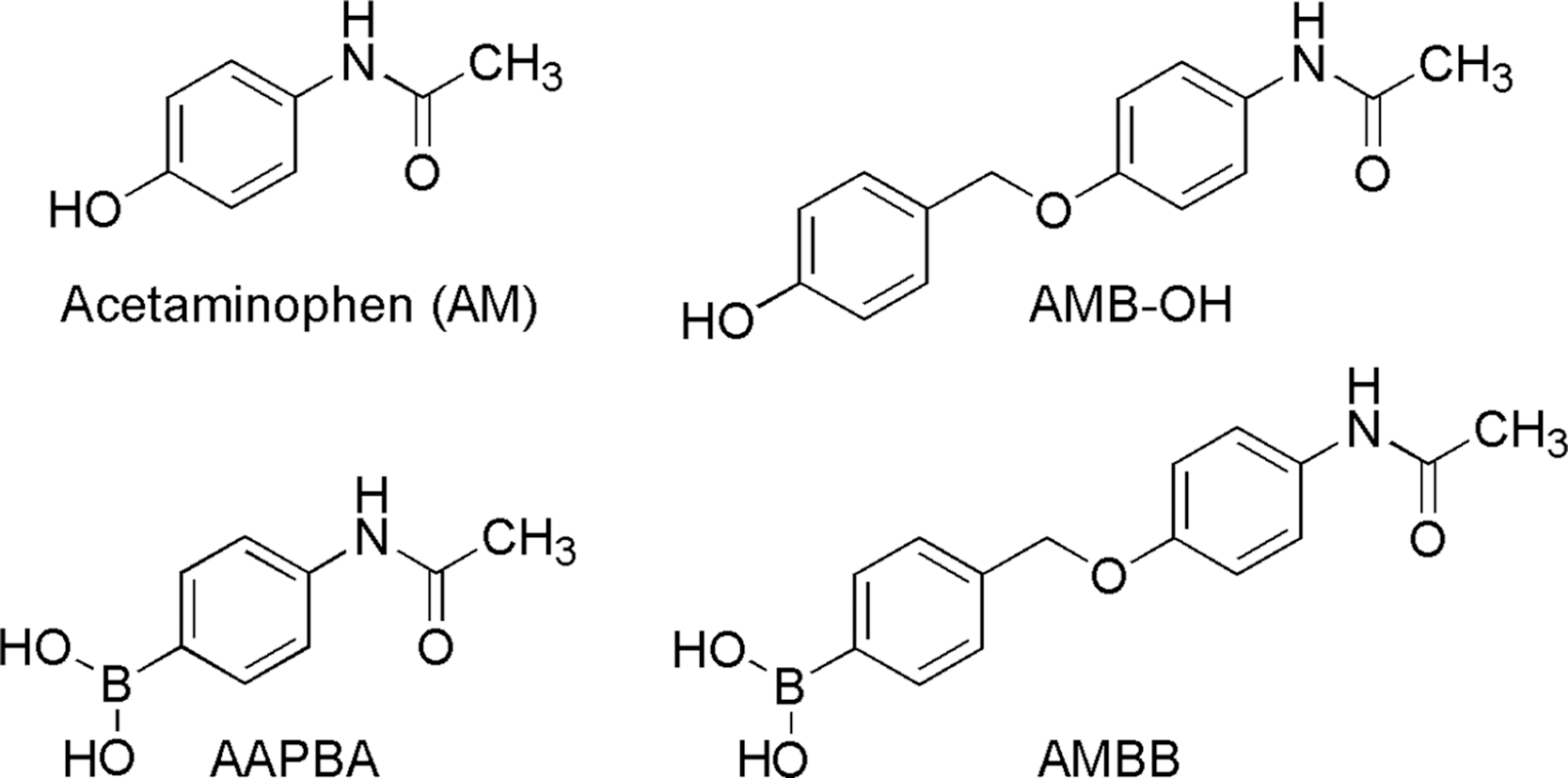
Chemical Structures of Acetaminophen (AM), *N*-[4-(4-Hydroxybenzyloxy)phenyl]acetamide (AMB-OH), 4-Acetamidophenylboronic Acid (AAPBA), and (4-((4-Acetamidophenoxy)methyl)phenyl)-boronic Acid, the Boronobenzyl Derivative of Acetaminophen (AMBB)

Acetaminophen [AM, *N*-(4-hydroxyphenyl)acetamide, also known as paracetamol or APAP] is a well-known analgesic and antipyretic drug and an active ingredient of hundreds of medicines used to relieve pain and fever.^14,15^ This simple phenolic compound is, like most other phenols, a substrate for heme peroxidases, and inhibition of heme peroxidases and oxygenases is believed to contribute to the beneficial effects of AM. It has been shown that AM is a substrate for compound I and compound II of cyclooxygenase-2 (COX-2), reducing this enzyme to its inactive form and stopping the production of prostaglandin E_2_.^15^ It also has been shown that AM is an inhibitor of MPO, switching it to a peroxidase cycle and inhibiting HOCl formation.^14^ This has been shown for both the isolated enzyme and stimulated human neutrophils at pharmacologically achievable concentrations. Despite all the well-documented beneficial effects of AM, its use and dosage are limited by the risk of severe liver injury. Hepatotoxicity of AM has been attributed to its oxidation by the P450 enzymes to the corresponding iminoquinone derivative, which can deplete cellular glutathione and modify cellular proteins.^15^ In order to limit its potential toxicity and to increase specificity to the sites of inflammation, we designed and synthesized a boronobenzyl derivative of acetaminophen, AMBB (Scheme 3). We anticipated that AMBB would lack the MPO inhibitory activity and show resistance to hepatic oxidation to the iminoquinone derivative, while oxidation of the arylboronic acid moiety of AMBB would lead to the formation of a phenolic derivative and subsequently to the release of AM via QM elimination during inflammation.^16^ We believe the proposed strategy may help establish a new class of COX and MPO pro-inhibitors that would release an active inhibitor only at the sites of inflammation, characterized by elevated levels of boronate-reactive oxidant(s).

In this study, the capability of AMBB to inhibit the MPO-derived HOCl production was evaluated and compared with AM and its structural analog 4-acetamidophenylboronic acid (AAPBA) (Scheme 3). Moreover, the reaction products of AMBB with HOCl and ONOO^–^ were identified by liquid chromatography–mass spectrometry (LC/MS), and the kinetics of the conversion of AMBB to AM was studied. The effect of uric acid and human serum albumin (HSA) on AMBB oxidation by ONOO^–^ and HOCl was also evaluated.

## Experimental Procedures

### Compounds

Acetaminophen, HSA, uric acid, H_2_O_2_, sodium hypochlorite, *N*,*N*,*N*′,*N*′-tetramethylbenzidine, sodium chloride, taurine, and 4-bromomethylphenylboronic acid were purchased from Sigma-Aldrich and were of the highest purity grade available. MPO from human neutrophils was obtained from Athens Research and Technology (Athens, GA). Peroxynitrite was synthesized and quantified according to the procedure described elsewhere.^17^ Ultrapure water (Millipore Integral 10, Millipore, MA) was used for the preparation of all aqueous solutions of the studied compounds.

### Syntheses

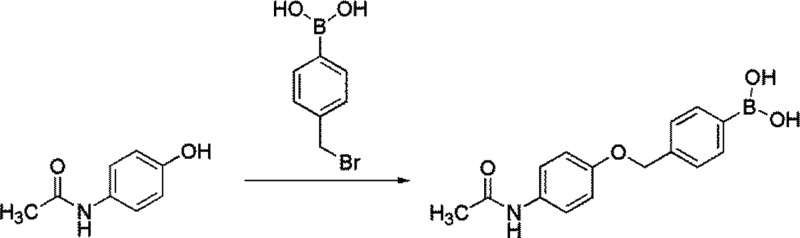



#### (4-((4-Acetamidophenoxy)methyl)phenyl)-boronic Acid (AMBB)

Acetaminophen (151 mg, 1 mmol), 4-bromomethylphenylboronic acid (215 mg, 1 mmol), and potassium carbonate (418 mg, 3 mmol, 3 equiv) were placed in a screw cap glass tube containing 2 cm^3^ of acetone. The mixture was stirred under reflux for 16 h. Then, the solvent was evaporated. The residue was placed on the filter funnel and washed several times with acetone. The precipitate was dried under reduced pressure for 2 h, giving 260 mg of the product with a yield of 91%. ^1^H NMR (CD_3_OD, 250.13 MHz, ppm): δ 7.59 (m, 2H), 7,40 (m, 4H), 6.93 (m, 2H), 5.05 (s, 2H), 2.09 (s, 3H) (Supplementary Figure S1). ^13^C NMR (CD_3_OD, 101 MHz, ppm) 170.04, 155.65, 133.33, 131.74, 126.20, 125.70, 122.01, 121.66, 114.71, 69.97, 47.68, 22.21 (Supplementary Figure S2). HRMS-ESI calculated for the formula C_15_H_16_^11^BNO_4_*m*/*z*[M + H^+^] = 286.1238, obtained *m*/*z*[M + H^+^] = 286.1251 (Supplementary Figures S3 and S4).

4-Acetamidophenylboronic acid was synthesized according to the procedure described in the Supporting Information (SI).

### LC/MS Measurements

Ultra-performance liquid chromatograph (UPLC) Acquity (Waters Ltd.) equipped with a photodiode array detector and combined with an LCT Premier XE (Waters Ltd.) mass spectrometer was used for the chromatographic separation and identification of AMBB and AAPBA oxidation products. Separation was performed on a reversed-phase C_18_ UPLC column (Waters Acquity UPLC BEH C_18_ 1.7 mm, 50 × 2.1 mm) equilibrated with a water/acetonitrile (CH_3_CN) (90/10 v/v) mobile phase containing 0.1% (v/v) trifluoroacetic acid (TFA). The analytes were separated at a flow rate of 0.3 mL/min. During the first 0.5 min of analysis, the composition of the mobile phase remained unchanged. The fraction of CH_3_CN was then gradually increased from 10 to 82% over the next 2 min. The injection volume was 2 μL, the sample temperature was 20 °C, and the column temperature was 40 °C. The electrospray source was operated at positive ion mode using the following parameters: capillary voltage 2.8 kV, sample cone voltage 60 V, desolvation temp. 350 °C, source temp. 100 °C, desolvation gas flow 800 L/h, and cone gas flow 50 L/h. The MCP detector voltage was 2.5 kV.

### Dose–Response Curves

The performance of AM and its derivatives in inhibition of the MPO chlorinating activity was evaluated using dose–response curves. The half maximal inhibitory concentration (IC_50_) values were determined on the basis of three independent measurements using a dose–response fitting mode implemented in OriginPro 2020 (OriginLab Corp.) software. The taurine *N*-chloramine/TMB assay and oxidation of the NBD-TM (4-thiomorpholino-7-nitrobenz-2-oxa-1,3-diazole) probe were used to detect the MPO-derived HOCl.^18−20^ MPO stock solution was prepared according to the supplier’s instruction by dissolving lyophilized powder of MPO in distilled water and was stored at 6 °C. The desired MPO activity was obtained by dilution of the stock solution with phosphate buffer (50 mM, pH 7.4) containing sodium chloride (0.1 M).

### Taurine *N*-Chloramine/TMB Assay

The diluted MPO solution was pipetted directly into the 96-well plate (1.45 μL). To the enzyme, 110 μL per well of solutions containing phosphate buffer (40 mM, pH 7.4), sodium chloride (0.2 M), taurine (40 mM), and the appropriate concentration of the studied compound were added. The prepared 96-well plate was incubated for 5 min, and the reaction was started by the addition of 110 μL per well of 20 μM H_2_O_2_ solution in water. The plate was incubated for another 5 min, and then 200 μL of the incubation mixtures was transferred to another 96-well plate prefilled with catalase solution (10 kU/mL, 2 μL per well) to remove unreacted hydrogen peroxide and to stop the reaction. Next, 50 μL per well of developing reagent composed of 2 mM TMB, 100 μM potassium iodide, and 0.32 M acetate buffer (pH 5.4) was added, and absorbance at 645 nm was measured by a Varioscan LUX (Thermo Fisher Scientific) plate reader controlled by SkanIt 6.0.2 software. The typical chlorinating activity of MPO during the inhibition experiments was 5 nM/s HOCl as determined using a CBA oxidation-based assay.^18^ The concentration of H_2_O_2_ in the stock solution was determined spectrophotometrically at 240 nm using the molar extinction coefficient ε = 43.6 M^–1^ cm^–1^. The chemical structures of the TMB and CBA probes, along with their oxidation products, are presented in Supplementary Figure S5.

### NBD-TM Assay

MPO-derived HOCl was monitored by an NBD-TM probe according to the previously described procedure (Supplementary Figure S5).^18^ Briefly, to 96-well plates containing MPO, NBD-TM (40 μM), the studied compounds, phosphate buffer (100 mM, pH 7.4), and sodium chloride (0.2 M), 20 μM solution of hydrogen peroxide was added to start the reaction (mixing ratio 1:1 v/v). Then, the changes of fluorescence intensity were measured by Varioscan LUX using λ_ex_ = 475 nm, λ_em_ = 575 nm, and slits width of 12 nm. This enabled the direct monitoring of fluorescent NBD-TSO formation.

### Kinetic Measurements

The rate constants of the reactions of AMBB and AAPBA with ONOO^–^ and HOCl were determined by a competition kinetic method using FLBA as a reference compound.^18,21,22^ In this approach, two pseudo-first-order reactions were assumed, a reaction between FLBA and the selected oxidant, ONOO^–^ or HOCl (eq 1), and between the investigated acetaminophen derivative (R-B(OH)_2_) and the oxidant (eq 2). Typically, the set of samples containing FLBA (20 μM), phosphate buffer (50 mM, pH 7.4), and AMBB or AAPBA (0–160 μM) in Eppendorf tubes was prepared. A concentrated basic solution of HOCl or ONOO^–^ was then added to the samples and rapidly mixed. The resulting concentration of an appropriate oxidant was 1.5 μM. Next, the samples were transferred to a 96-well plate, and the fluorescence intensity of the formed fluorescein was measured. The fluorescence intensity was followed with a Varioscan LUX plate reader using λ_ex_ = 490 nm, λ_em_ = 515 nm, and slit widths of 12 nm. Using the concentration ratios of fluorescein (product of oxidation of FLBA), [FL]_0_/[FL], where [FL]_0_ is the yield of fluorescein in the absence of the acetaminophen derivative, and the previously reported rate constants for the FLBA reactions with ONOO^–^ (^2^*k* = 1.0 × 10^6^ M^–1^ s^–1^)^21^ and HOCl (^2^*k* = 1.11 × 10^4^ M^–1^ s^–1^),^18^ the rate constants for the reaction of AMBB and AAPBA with both oxidants were calculated according to eq 3.
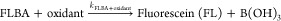
1
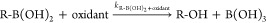
2
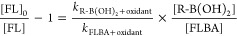
3

The rate constants for H_2_O_2_ and the studied boronic acids were determined directly under the pseudo-first-order conditions. Typically, the incubation mixtures contained boronic acid (20 μM), phosphate buffer (20 mM), CH_3_CN (2.5% v/v), and at least 10-fold excess of H_2_O_2_ (0.2–1 mM). The changes of analyte concentrations were followed by UPLC using a photodiode array detector (λ_max_ = 250 ± 1.2 nm). On the basis of kinetic traces of AMBB and AAPBA decay (Supplementary Figure S6), the pseudo-first-order rate constants were determined. The second-order rate constants were calculated using the linear relationship between the determined pseudo-first-order rate constants and H_2_O_2_ concentrations.

### UPLC Analyses of AMBB/HSA-Containing Samples

Prior to UPLC analyses of HSA-containing samples, the protein was precipitated by mixing (1:1 v/v) with an ice-cold solution of 0.1% (v/v) TFA in methanol. Then, the samples were vortexed and centrifuged (20,000 × *g*, for 30 min at 4 °C). The obtained supernatants were diluted (1:1 v/v) with a 0.1% (v/v) ice-cold aqueous TFA solution and centrifuged again (20,000 × *g*, for 15 min at 4 °C). Such prepared samples were analyzed by UPLC.

## Results

### Inhibition of MPO-Derived HOCl Production by AM Derivatives

**Figure 1:**
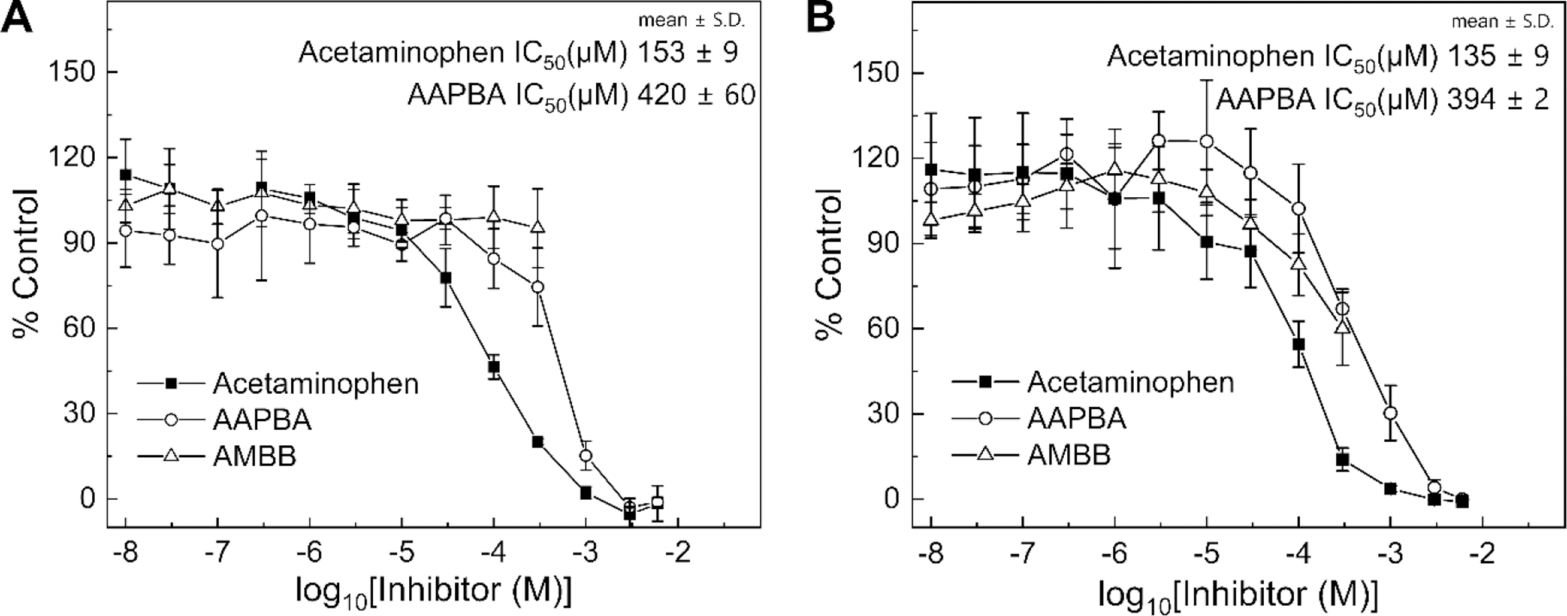
Dose–response curves of MPO inhibition for acetaminophen, AMBB, and AAPBA determined by the taurine *N*-chloramine/TMB assay (A) and NBD-TM oxidation (B). Mixtures contained taurine (20 mM) or NBD-TM (20 μM), MPO (0.1 nM, 5 nM HOCl/s), H_2_O_2_ (10 μM), NaCl (0.1 M), phosphate buffer (20 or 50 mM, pH 7.4), 3% (v/v) methanol, and the appropriate compound AM (0.01 μM–6 mM), AMBB (0.01–300 μM), or AAPBA (0.01 μM–6 mM). The experimental points were read for the incubation time of 5 min. Each panel is a representative result of three independent experiments and points represent means ± S.D.

The MPO inhibitory activity of AM was compared with its two synthesized boronated derivatives (Figure 1). The production of HOCl by MPO was monitored using two different assays: taurine *N*-chloramine-mediated TMB oxidation and direct oxidation of an NBD-TM probe by HOCl.^18−20^ In the taurine-based assay, HOCl oxidizes taurine to taurine *N*-chloramine. The resulting *N*-chloramine is then quantified in a reaction with *N*,*N*,*N*′,*N*′-tetramethylbenzidine catalyzed by potassium iodide.^20^ In the case of the NBD-TM probe, in the presence of HOCl, the sulfur atom in the probe is oxidized to sulfoxide, producing a highly fluorescent NBD-TSO.^18^

The dose–response curves for AM, AMBB, and AAPBA, obtained using a taurine-based assay, are shown in Figure 1A. For AMBB, in the range of 0 nM to 0.3 mM, no inhibition of HOCl production was observed (Figure 1A). The course of dose–response curve above 0.3 mM could not be determined due to limited solubility and precipitation of AMBB. For AAPBA and acetaminophen, the determined IC_50_ values are equal to 420 ± 60 and 153 ± 9 μM, respectively. Similar dose–response curves were obtained using the NBD-TM probe (Figure 1B), and the determined IC_50_ values for AAPBA and AM are 394 ± 2 and 135 ± 9 μM, respectively. Interestingly, in the case of the NDB-TM assay, AMBB shows the inhibitory effects on the HOCl production activity of MPO when used in the 0.1–0.3 mM concentration range (Figure 1B).

Overall, these experiments provide proof-of-principle confirmation that boronation of the hydroxyl phenolic group in AM, either directly or via boronobenzylation, decreases its MPO inhibitory potential. As AMBB showed lower inhibitory activity, as compared with AAPBA, in case of taurine chlorination, this compound was taken for further characterization of its oxidative conversion into AM.

### Identification of AMBB and HOCl Reaction Products

**Figure 2:**
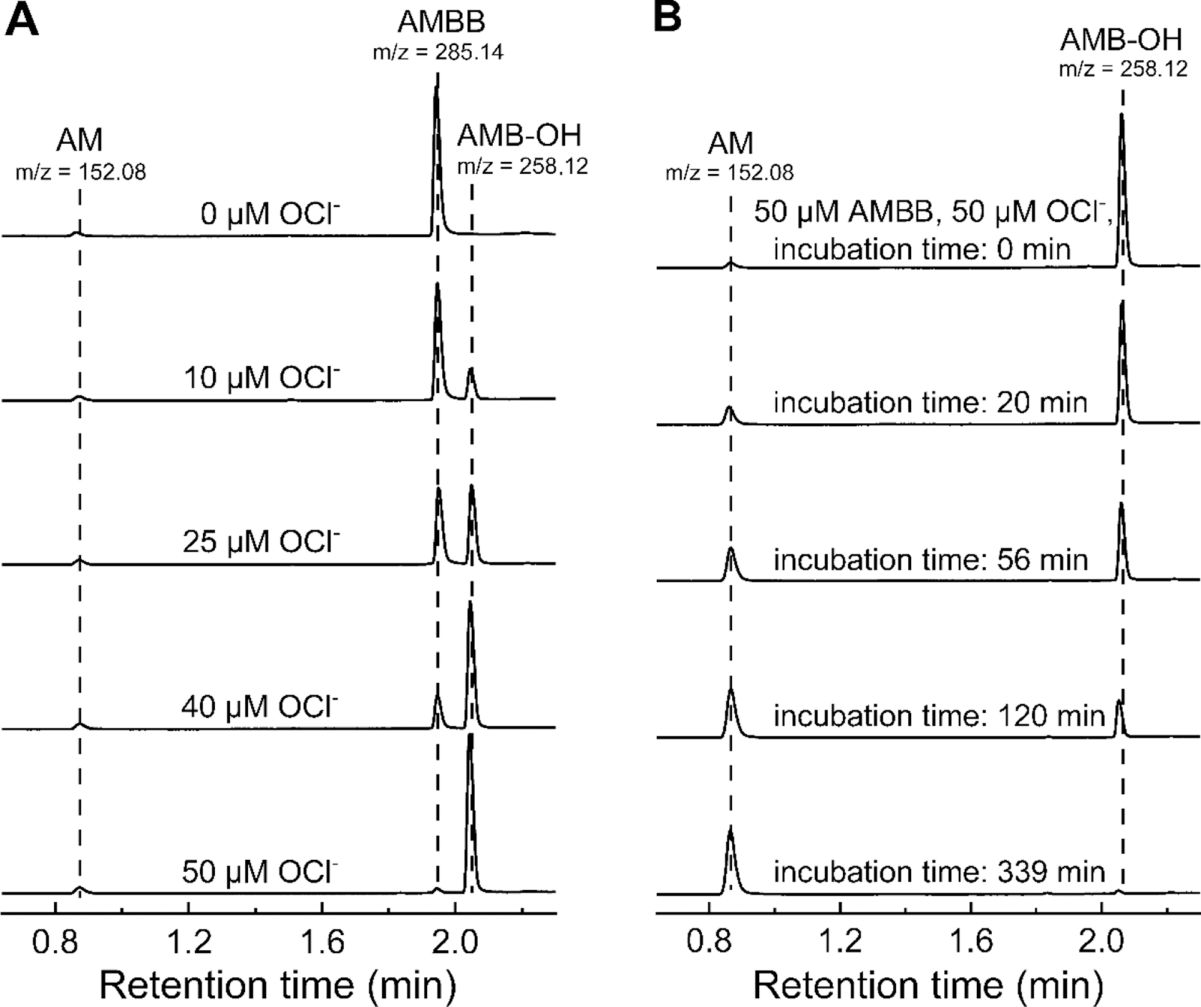
UPLC-MS analyses of AMBB and HOCl reaction products. (A) UPLC traces of samples containing 50 μM AMBB, various concentration of HOCl (0–50 μM), and 20 mM phosphate buffer (pH 7.4). Samples were analyzed immediately (<2 min) after mixing. (B) Spontaneous conversion of AMB-OH to acetaminophen (AM). Incubation mixture contained 50 μM AMBB, 50 μM HOCl, and 20 mM phosphate buffer. UPLC traces were extracted at 250 ± 1.2 nm. *m*/*z* values for [M + H]^+^ ions were obtained using a TOF-MS detector (see Supporting Information). Each panel is a representative result of three independent experiments.

**Figure 3:**
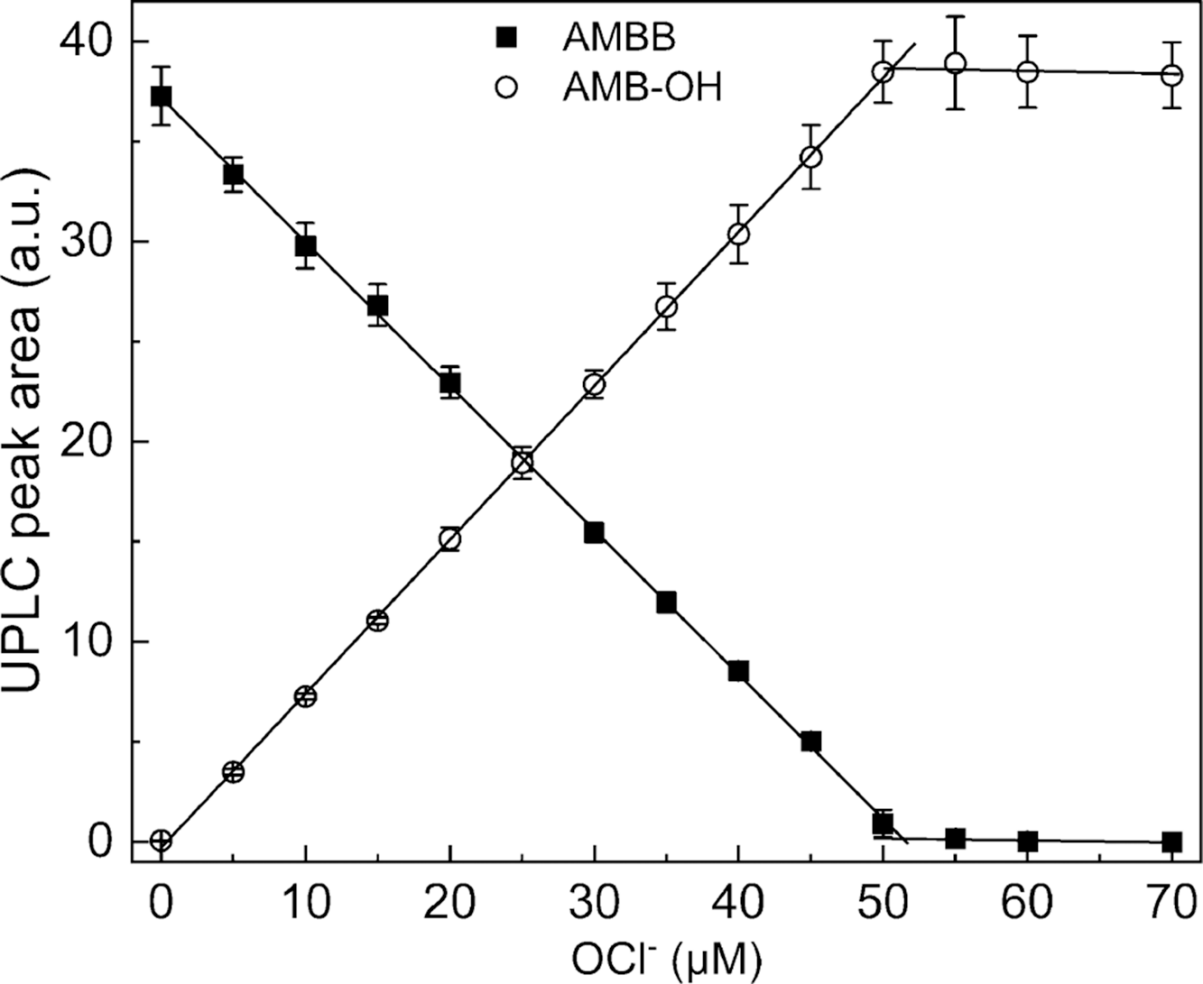
Stoichiometry of the AMBB and HOCl reaction. UPLC peak areas of AMBB (solid squares, 50 μM) and AMB-OH (open circles) as a function of sodium hypochlorite concentration in aqueous phosphate buffer (20 mM, pH 7.4). UPLC peak areas were integrated for chromatograms extracted at 250 ± 1.2 nm. Points represent means ± S.D. for three independent measurements.

To test the feasibility of the oxidative conversion of the AMBB proinhibitor into AM, AMBB was reacted with selected inflammatory oxidants. Products of the reaction between AMBB and HOCl were identified by UPLC with ultraviolet absorption and mass spectrometric detection (Figure 2). The bolus addition of sodium hypochlorite to a buffered solution of AMBB resulted in the formation of a new product eluting at 2.1 min (Figure 2A) with the molecular mass corresponding to the primary phenolic product *N*-[4-(4-hydroxybenzyloxy)phenyl]acetamide (AMB-OH) (Scheme 3, Supplementary Figure S7). No significant formation of AM was observed when the reaction mixture was analyzed immediately (<2 min) after mixing when the probe consumption already occurred. A stoichiometric analysis of AMBB consumption and AMB-OH formation indicates that both the decrease in the AMBB peak intensity and the build-up of the AMB-OH signal are proportional to the amount of HOCl added to the samples (Figure 3).

**Figure 4:**
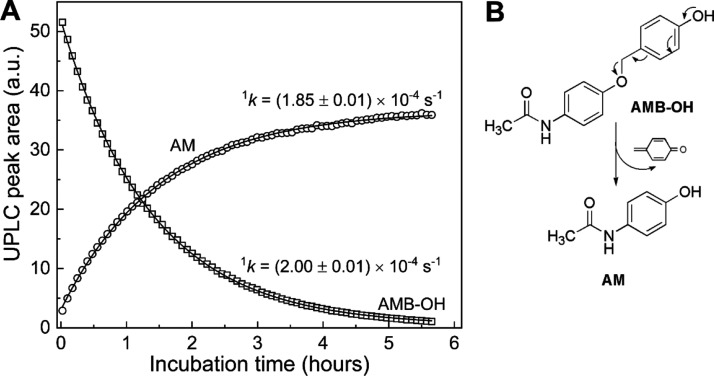
Kinetics of the conversion of AMB-OH into acetaminophen (AM). AMB-OH was produced in situ by mixing 50 μM AMBB with 50 μM NaOCl in 20 mM phosphate buffer, pH 7.4. (A) UPLC peak areas of AMB-OH (open squares) and AM (open circles) as a function of incubation time. UPLC peak areas were integrated for chromatograms extracted at 250 ± 1.2 nm. (B) Scheme of the quinone methide moiety elimination by AMB-OH to form AM.

**Figure 5:**
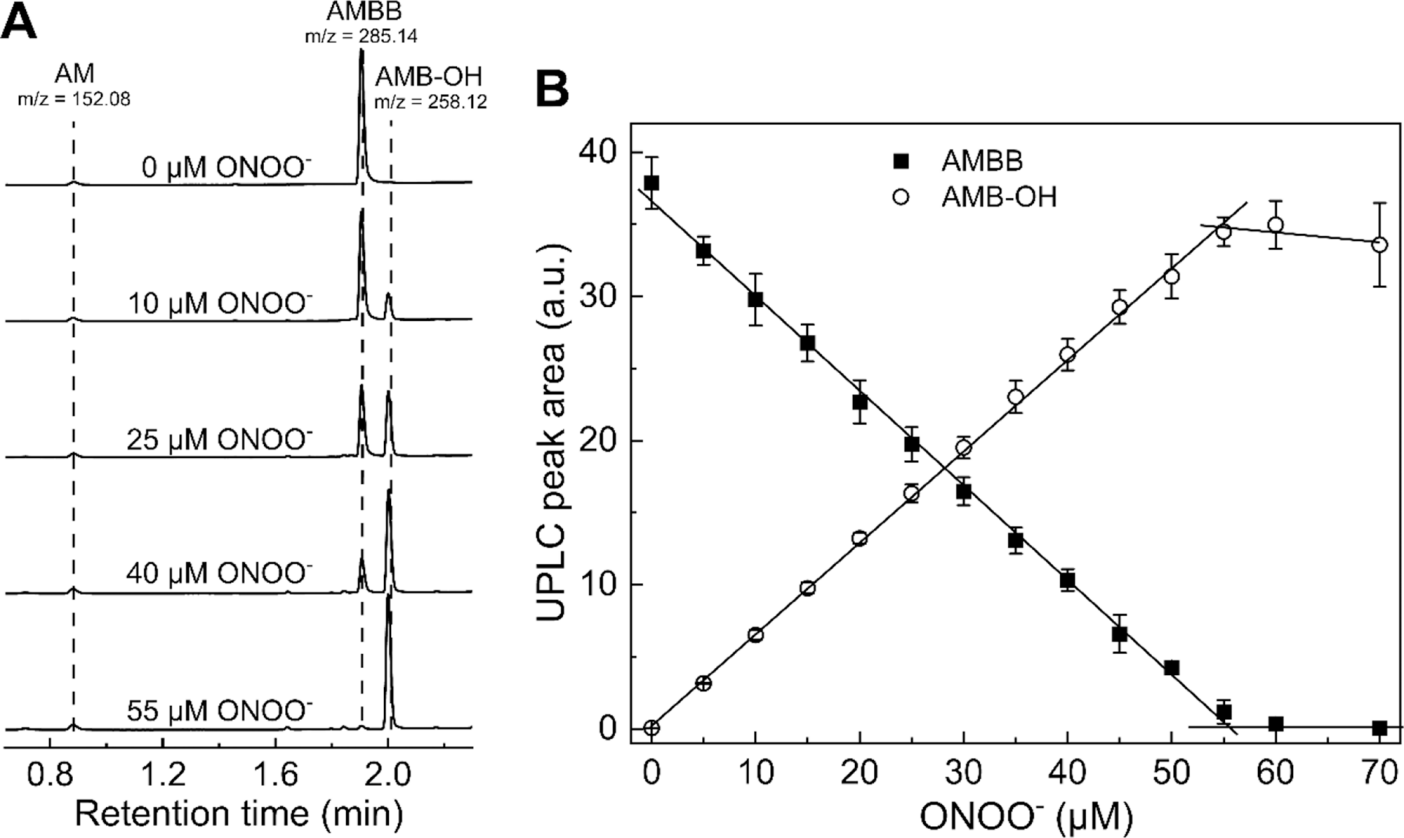
UPLC-MS analyses of the AMBB and ONOO^–^ reaction mixtures. (A) UPLC traces of samples containing 50 μM AMBB, various concentrations of ONOO^–^ (0–50 μM), and 20 mM phosphate buffer (pH 7.4). Samples were analyzed immediately (<2 min) after mixing. UPLC traces were extracted at 250 ± 1.2 nm. *m*/*z* values for [M + H]^+^ ions were obtained using a TOF-MS detector (see Supporting Information). (B) Stoichiometry of AMBB and ONOO^–^ reaction. UPLC peak areas of AMBB (solid squares, 50 μM) and AMB-OH (open circles) as a function of ONOO^–^ concentration in aqueous phosphate buffer (20 mM, pH 7.4). UPLC peak areas were integrated for chromatograms extracted at 250 ± 1.2 nm. Points represent means ± S.D. for three independent measurements.

The addition of an equimolar amount of HOCl to AMBB solution completely abolished the AMBB peak. Addition of excess of HOCl to the incubation mixture resulted in a decay of the AMB-OH peak, suggesting the AMB-OH product reacts with excess HOCl. Prolonged incubation of the reaction mixture in the buffered solution led to the decomposition of the primary phenolic product, AMB-OH, accompanied by the build-up of a new peak with a retention time of 0.87 min and a mass of 152.08 Da (Figure 2B), which corresponds to the retention time and mass of the authentic standard of AM. This confirms that AM is an ultimate product of the oxidation of AMBB by HOCl. Kinetic analysis of the transformation of AMB-OH into AM (Figure 4) indicates a first-order process with the value of the rate constant of 2.0 × 10^–4^ s^–1^, which corresponds to a half-life time of AMB-OH of 1 h at pH 7.4 and room temperature, with a complete conversion occurring only after 5 h of incubation. Overall, the presented results indicate that AMBB reacts rapidly with HOCl in a stochiometric ratio 1:1, producing AMB-OH as the primary product that, within hours of incubation, subsequently undergoes spontaneous transformation to AM. Similar results were obtained for the reaction of AMBB with ONOO^–^, with AMB-OH formed as the major product and AM formed in only small yield immediately after mixing (Figure 5). Similar to the reaction with HOCl, AMBB reacts with ONOO^–^ with a 1:1 stoichiometry. Also, in the case of H_2_O_2_, the reaction with AMBB leads to the formation of AMB-OH, which upon prolonged incubation undergoes spontaneous transformation to AM under the conditions used (Supplementary Figure S8).

### Kinetics of the Reaction of AMBB and AAPBA with Inflammatory Oxidants

**Figure 6:**
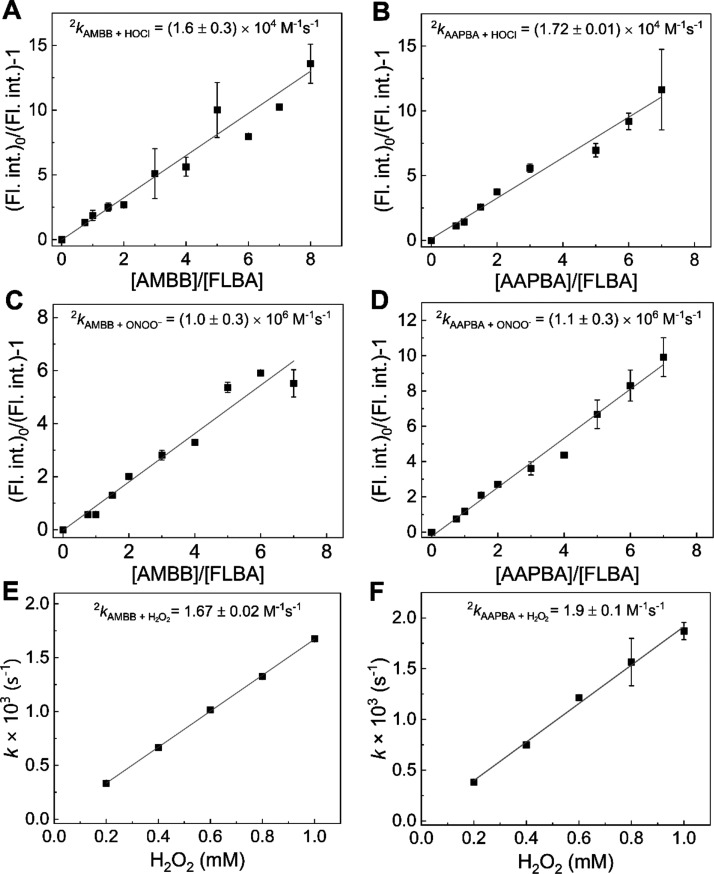
Kinetics of the reaction between the boronate derivatives of acetaminophen and HOCl, ONOO^–^, and H_2_O_2_, determined at pH 7.4. (A) Relationship used to determine the rate constant between AMBB and HOCl according to the competition kinetic approach. Incubation mixtures contained 20 μM FLBA, 15–160 μM AMBB, 1.5 μM NaOCl, 50 mM phosphate buffer (pH 7.4), and 5% (v/v) ACN. (B) Same as (A) but incubation mixtures contained AAPBA instead of AMBB. (C) Same as (A) but incubation mixtures contained 1.5 μM ONOO^–^ instead of NaOCl. (D) Same as (B) but incubation mixtures contained 1.5 μM ONOO^–^ instead of NaOCl. (E) Dependence of pseudo-first-order rate constant of AMBB oxidation on a H_2_O_2_ concentration. Incubation mixtures contained 0.2–1 mM H_2_O_2_, 20 μM AMBB, 20 mM phosphate buffer (pH 7.4), and 2.5% (v/v) ACN. (F) Same as (E) but incubation mixtures contained AAPBA instead of AMBB. The solid lines represent the linear fittings according to the presented equation (see the Experimental Procedures section). Points represent means ± S.D. for at least two independent measurements.

The rate constants of the reaction between AMBB and the biologically relevant two-electron oxidants, HOCl, ONOO^–^, and H_2_O_2_, were determined (Figure 6A,C,E). The values of the rate constants are equal to (1.6 ± 0.3) × 10^4^, (1.0 ± 0.3) × 10^6^, and 1.67 ± 0.02 M^–1^ s^–1^ for the reactions with HOCl, ONOO^–^, and H_2_O_2_, respectively. These values suggest that HOCl and ONOO^–^ are the most likely biological oxidants to efficiently oxidize AMBB and produce AM. For the second derivative of acetaminophen, AAPBA, the determined rate constants are of similar magnitude and are equal to (1.72 ± 0.01) × 10^4^ M^–1^ s^–1^ for HOCl, (1.1 ± 0.3) × 10^6^ M^–1^ s^–1^ for ONOO^–^, and 1.9 ± 0.1 M^–1^ s^–1^ for H_2_O_2_ (Figure 6B,D,F).

### Effect of Uric Acid and HSA on the Extent of AMBB Oxidation by ONOO^–^, HOCl, and H_2_O_2_

**Figure 7:**
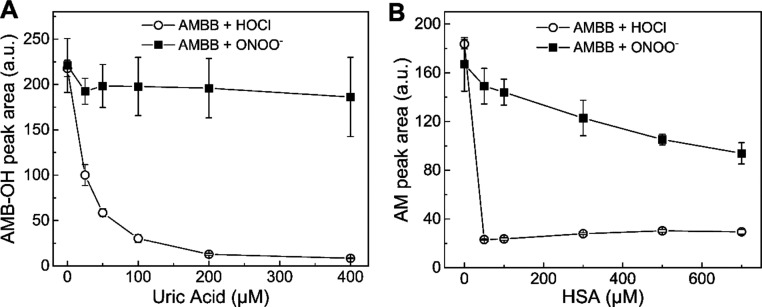
Effect of uric acid and HSA on the extent of oxidation of AMBB by HOCl and ONOO^–^. (A) UPLC peak areas of AMB-OH. Incubation mixtures contained 100 μM AMBB, 50 μM HOCl, or ONOO^–^, 0–400 μM uric acid, 20 mM phosphate buffer (pH 7.4), and 5% (v/v) ACN. Samples were analyzed immediately after mixing. (B) UPLC peak areas of acetaminophen. Incubation mixtures contained 100 μM AMBB, 50 μM HOCl, or ONOO^–^, 0–700 μM HSA, 20 mM phosphate buffer (pH 7.4), and 5% (v/v) ACN. Samples were analyzed 24 h after mixing. UPLC peak areas were integrated for chromatograms extracted at 250 ± 5 nm. Points represent means ± S.D. for at least two independent measurements.

In vivo application of AMBB as a proinhibitor of heme proteins (e.g., MPO and COX-2) requires efficient oxidation of the compound in the presence of biological competitors/scavengers of the oxidants. Two potential antioxidant scavengers of HOCl and ONOO^–^ in human plasma are HSA and uric acid.^23−26^ Thus, the influence of uric acid and HSA, at their physiologically relevant concentrations,^27,28^ on the reaction of AMBB with HOCl and ONOO^–^ was investigated (Figure 7). For this purpose, a set of samples containing 100 μM AMBB, from 0 to 400 μM uric acid, 20 mM phosphate buffer (pH 7.4), and 5% acetonitrile was prepared. Then, a concentrated HOCl solution was added to each sample, and the produced AMB-OH was determined by UPLC (Figure 7A and Supplementary Figure S9). In the absence of uric acid, the distinct peak of AMB-OH was visible (Supplementary Figure S9A). The presence of 25 μM uric acid in the sample reduced the amount of AMB-OH by approximately half, and almost complete inhibition of AMBB oxidation was observed in the presence of 200 μM uric acid (Figure 7A). A similar experiment using ONOO^–^ as the oxidant indicates that uric acid has virtually no effect on the extent of AMBB oxidation (Figure 7A and Supplementary Figure S9B). The UPLC traces measured after an extended incubation time are also available in the SI (Supplementary Figure S9C,D), allowing for monitoring the yield of the final product of oxidation, AM, and expectedly show the same trends. Similar experiments were performed for AMBB oxidation in the presence of HSA at concentrations of 25 to 700 μM (Figure 7B and Supplementary Figure S10). Prior to UPLC analysis, HSA had to be precipitated. So in this case, the amounts of AM formed after extended incubation time were determined. For samples containing 100 μM AMBB, 20 mM phosphate buffer (pH 7.4), and 5% acetonitrile, the addition of 50 μM HOCl resulted in the formation of 50 μM AM. The presence of any amount of HSA, for instance, 25 μM, completely abolished the peak of AM (Figure 7A and Supplementary Figure S10A). For incubations where ONOO^–^ was used as the oxidant, the effect of the presence of HSA was much less pronounced and at the highest HSA concentration tested (700 μM), the yield of AM was decreased to about 50% (Figure 7B and Supplementary Figure S10B). These data strongly suggest that in biological systems, where endogenous scavengers may completely prevent oxidation of AMBB by HOCl, the compound still may be efficiently oxidized by ONOO^–^ to generate AM at the site of inflammation.

The effect of the presence of uric acid and HSA on H_2_O_2_-induced oxidation of AMBB was studied under analogous conditions. Uric acid did not affect the yield of the AM oxidation product, but HSA significantly reduced the efficiency of AM formation (Supplementary Figure S11). Nonetheless, given the normal plasma concentration of H_2_O_2_ of approximately 1–5 μM and up to 30–50 μM under inflammatory conditions,^29^ and the determined AMBB/H_2_O_2_ reaction rate constant (*k* = 1.67 ± 0.02 M^–1^ s^–1^), the AMBB/H_2_O_2_ reaction is not expected to significantly contribute to AMBB oxidation in vivo. Moreover, at sites of inflammation, MPO would scavenge H_2_O_2_, potentially depleting the pool of this oxidant.

## Discussion

Acetaminophen has been shown to inhibit MPO-catalyzed HOCl production at therapeutically achievable concentrations.^14^ The mechanism of inhibition of MPO-dependent HOCl production by AM is related to diverting the enzyme from the halogenation cycle to oxidation of AM in the peroxidase cycle. In order to modulate the capability of AM to inhibit HOCl production by MPO, we synthesized its new boronobenzyl derivative substituted with an arylboronic acid moiety, AMBB. The results obtained by two independent assays indicate that the substitution of AM with a boronate or boronobenzyl moiety decreases its ability to inhibit the MPO halogenation cycle (Figure 1). The observed inhibitory effects of AMBB and AAPBA cannot be the result of a direct reaction of the compounds with HOCl because even at the highest concentration of AAPBA (6 mM) the rate of taurine chlorination is still more rapid than AAPBA/HOCl reaction. The differences in the activity of AM and its boronate derivatives are likely due to the different reactivity of these compounds with compound I and compound II. The electrode potential at pH 7 for AM (AM-OH/AM-O^·^,H^+^ redox couple) is estimated to be *E*°′ = 0.71 ± 0.01 V.^30^ This potential is much lower than for the redox couples of species involved in the peroxidase cycle of MPO (*E*°′_compound I/compound II_ = 1.35 V; *E*°′_compound II/ferric enzyme_ = 0.97 V);^7,31,32^ thus, AM may be very easily oxidized by compounds I and II of MPO. The electrode potential for the AAPBA/AAPBA^·+^ redox couple is expected to be significantly higher than for the AM-OH/AM-O^·^,H^+^ redox couple. This assumption is supported by the Hammett constants (σ_p_) that are equal to −0.37 for the hydroxyl substituent (AM) and 0.12 for the boronic acid moiety.^33^

In the presence of taurine, AMBB turned out to not show any inhibitory activity toward the MPO halogenation cycle at the concentration up to 0.3 mM. The explanation of this phenomenon may lie in the chlorination mechanism of taurine. Studies on MPO-catalyzed taurine chlorination have shown that taurine can form *N*-chlorotaurine during the direct reaction with HOCl (Reaction R1) and/or via the reaction with a chlorinating compound one/chloride anion complex inside the heme pocket (Reaction R2).^34^
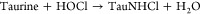
R1
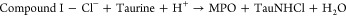
R2

Consequently, the size of the substrate and the steric hindrance of its substituents play a role in the chlorination mechanism. According to what has been suggested by Ramos et al., small substrates such as taurine will be chlorinated inside and outside the enzyme (Reactions R1 and R2) and bulkier ones only outside (Reaction R1).^35^ In the experiment where AMBB and taurine are present, there is a competition between Reactions R2 and R3. However, Reaction R2 is much more favorable than Reaction R3, as confirmed by the experiment (Figure 1A), and the steric hindrance of AMBB may play a role. Definitely, the key point is that taurine undergoes chlorination reaction inside the heme pocket.
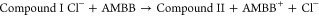
R3

In the experiment where both AMBB and taurine were present, taurine was chlorinated inside the heme pocket, and bulky AMBB was not able to effectively compete with taurine for compound I. Therefore, AMBB failed to inhibit the chlorination cycle in the presence of taurine. Analogical results were obtained for incubations containing lower concentrations of taurine, ranging from 0.1 to 20 mM (Supplementary Figure S12). The concentration of taurine in the plasma and interstitial fluids ranged from 10 to 100 μM, and, depending on the cell type, the intracellular concentration of taurine reached up to 50 mM.^36,37^ Therefore, it is reasonable to conclude that AMBB in the presence of physiological concentrations of taurine will not inhibit the MPO chlorination cycle.

This explanation is further supported by the results obtained for the NBD-TM probe (Figure 1B). In the absence of taurine, AMBB—like other AM derivatives—exhibits inhibitory activity toward the MPO chlorination cycle.

Furthermore, the inhibition of taurine chlorination as a result of a direct reaction of AMBB with HOCl can be excluded, comparing the taurine chlorination rate constant (^2^*k* = 4.8 × 10^5^ M^–1^ s^–1^)^38^ with the AMBB/HOCl rate constant (^2^*k* = 1.6 × 10^4^ M^–1^ s^–1^), as well as the concentration of taurine (20 mM), and AMBB (300 μM) used in the experiment (Figure 1A).

The LC/MS study of the reaction products demonstrated that AMBB reacts with HOCl to form the corresponding phenol derivative (AMB-OH) (Figure 2A). In contrast with many other reported benzyloboronate derivatives of phenolic compounds, the primary phenol formed upon oxidation of AMBB is relatively stable and undergoes spontaneous transformation with the elimination of the QM moiety only after extended incubation (minutes to hours).^16^ The LC/MS experiment has also shown that upon QM elimination, AMB-OH is converted into the AM molecule (Figure 2B). The rate constants of the reactions of AMBB with selected inflammatory oxidants (HOCl, ONOO^–^, and H_2_O_2_) have been determined (Figure 6), and these values are of the same order of magnitude as for other boronic acids and esters.^9,10,16,39^

1,4-Quinone methide (QM), released by AMBB, is an unsaturated carbonyl compound that can react with nucleophiles.^40^ Zielonka et al. have shown that in an aqueous solution, QM reacts with water to form 4-hydroxybenzyl alcohol,^10^ which is a harmless, naturally occurring compound.^41^ In plasma, where water is the main component, this reaction may be the major pathway of QM decay. However, other nucleophiles, including thiols, are also expected to react with QM, especially inside cells, where multiple nucleophiles are much more abundant. Thus, if produced in large amounts, released QM is expected to exhibit cytotoxic effects.

**Scheme 4:**
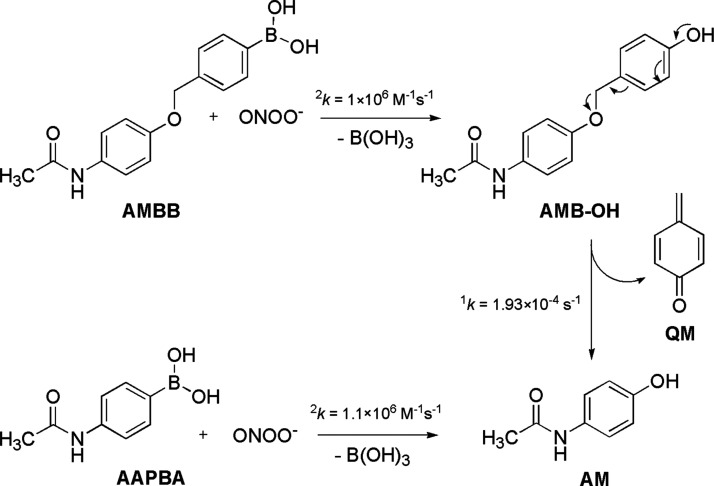
Mechanism of Peroxynitrite-Induced Quinone Methide Elimination by AMBB

Finally, we investigated the effect of uric acid and HSA, the ubiquitous plasma components,^27,28^ on the oxidation of AMBB by HOCl and ONOO^–^ (Figure 7), the oxidants produced at sites of inflammation.^42^ We have observed that both uric acid and HSA show strong inhibitory effects on the oxidation of AMBB by HOCl (Figure 7). Taking into account the presented results, the bimolecular rate constant for the reaction of HOCl with uric acid (3 × 10^5^ M^–1^ s^–1^, pH 7),^43−45^ and the fact that HSA is the main scavenger of HOCl at sites of infection and inflammation,^23−26^ under physiological conditions, there seems to be very little chance to release the active form of inhibitor via the reaction of HOCl with AMBB. However, an exception to such conclusion may be the highly oxidizing environment of the phagosomes. On the other hand, uric acid does not affect the AMBB/ONOO^–^ reaction, and AMBB is converted to AM even in the presence of physiological concentrations of HSA up to 700 μM (Figure 7B).^27^ In the case of AMBB/ONOO^–^ reaction, in addition to the effect of HSA, the effect of CO_2_ should be considered because ONOO^–^ reacts with CO_2_, forming a nitrosoperoxycarbonate anion (ONOOCO_2_^–^) that decomposes rapidly to ^•^NO_2_ and CO_3_^•–^. Nonetheless, Sikora et al. have shown that in the presence of a high concentration of HCO_3_^–^_,_ boronic acids are still efficiently converted into corresponding phenols.^9^ Overall, these results suggest that under physiological conditions, ONOO^–^ may act as the major oxidant that can oxidize AMBB and release AM (Scheme 4).

During the course of this project, we have identified three major limitations of the proposed design, as listed below.

1. Peroxynitrite seems to be the most likely activator of AMBB in biological systems. This would limit application of the boronate-based proinhibitors mostly to the conditions associated with elevated production of ONOO^–^.
2. Due to relatively high IC_50_ values for the inhibition of MPO and COX-2 by AM (150 and 25 μM, respectively), AMBB should demonstrate a high peak plasma concentration similar to that of AM. In addition, large amounts of the oxidant must be intercepted by AMBB to generate a sufficient amount of AM to inhibit the inflammatory processes. Future design should focus on applying the same strategy to more potent inhibitors, ideally with IC_50_ values in the nanomolar range.
3. The primary phenolic product of AMBB oxidation, AMB-OH, undergoes transformation to AM at an unexpectedly slow rate, with a half-life time of ca. 1 h at room temperature and pH 7.4. While this may be sufficiently fast, in comparison with the treatment of inflammation in patients, this introduces the possibility that AMB-OH may be subject to cellular metabolism without generation of AM. Therefore, future study design may require modification of the benzyl linker to accelerate the elimination of QM from AMB-OH.

## Conclusions

The described characteristics of the AMBB proinhibitor provide proof-of-concept for the design of oxidant-activated anti-inflammatory agents that would show inhibitory effects only under the conditions of the elevated levels of the oxidants. This is expected to limit the potential off-target effects and toxicity of the inhibitors. An additional value of the boronate-based agents is their ability to scavenge ONOO^–^ and prevent ONOO^–^-dependent oxidation and nitration processes. The potential synergy between scavenging ONOO^–^ by AMBB, and the analgesic activity of the reaction product, AM, is an exciting avenue to explore for the treatment of neuropathic pain.^46−49^

## Supporting Information Available

Description of AAPBA synthesis; ^1^H NMR, ^13^C NMR, and mass spectra of AMBB; chemical structures of TMB, CBA, COH, NBD-TM, and NBD-TSO compounds; kinetic traces of AMBB and AAPBA decay; mass spectrum of AMB-OH; UPLC chromatograms of AMBB and H_2_O_2_ incubation mixture; UPLC chromatograms of AMBB/HOCl/uric acid and AMBB/ONOO/uric acid incubation mixtures; UPLC chromatograms of AMBB/HOCl/HSA and AMBB/ONOO/HSA incubation mixtures; effect of uric acid and HSA on oxidation of AMBB by H_2_O_2_; and dose–response curves for AMBB determined by the taurine *N*-chloramine/TMB assay (PDF)
