# Supplementary material for: Boronate-Based Oxidant-Responsive Derivatives of Acetaminophen as Proinhibitors of Myeloperoxidase
Source: Chem Res Toxicol. 2023 Aug 3;36(8):1398–408. doi: 10.1021/acs.chemrestox.3c00140 (PMC10445283; doi:10.1021/acs.chemrestox.3c00140)
Supplement: Supplementary file 1 — tx3c00140_si_001.pdf [file tx3c00140_si_001.pdf]

Boronate-based oxidant-responsive derivatives of acetaminophen as proinhibitors of myeloperoxidase

Karolina Pierzchała,[1] Jakub Pięta,[1] Marlena Pięta,[1] Monika Rola,[1] Jacek Zielonka,[2,*] Adam Sikora,[1] Andrzej Marcinek[1], Radosław Michalski,[1,*]

[1]Institute of Applied Radiation Chemistry, Department of Chemistry, Lodz University of Technology, Zeromskiego 116, 90-924 Lodz, Poland

[2]Department of Biophysics and Free Radical Research Center, Medical College of Wisconsin, 8701 Watertown Plank Road, Milwaukee, WI 53226, United States

Corresponding author

*Radosław Michalski, PhD

Tel.: +48426313097

E-mail: radoslaw.michalski@p.lodz.pl

*Jacek Zielonka, PhD, DSc

Tel.: 001 414 955 4789

E-mail: jzielonk@mcw.edu

SUPPLEMENTARY INFORMATION

Table of Contents

Synthesis of AAPBA.. 3

Supplementary Figure S1.. 4

Supplementary Figure S2.. 5

Supplementary Figure S3.. 6

Supplementary Figure S4.. 7

Supplementary Figure S5.. 8

Supplementary Figure S6.. 8

Supplementary Figure S7.. 9

Supplementary Figure S8.. 10

Supplementary Figure S9.. 11

Supplementary Figure S10.. 12

Supplementary Figure S11.. 13

Supplementary Figure S12.. 13

Synthesis of 4-acetamidophenylboronic acid (AAPBA)

Acetaminophen (250 mg, 1.6 mM) was placed in a round-bottom flask filled with 5 cm^3 of anhydrous DCM under argon. After 10 min of purging with argon, DIPEA (575 µL, 3.3 mM, 2 eq) was added *via* syringe, and the mixture was placed in an ice-water bath. Finally, after cooling down, *N*-phenyl-bis(trifluoromethanesulfonimide) (650 mg, 1.82 mM, 1.1 eq) was added, and the reaction was stirred overnight warming gradually to room temperature. At this time point, TLC (95:5 DCM:MeOH, v/v) showed no traces of starting material. 15 cm^3 of 10% aqueous HCl solution was added, and the mixture was extracted with 3 × 20 cm^3 of EtOAc. Combined organic layers were washed with brine and dried with Na_2SO_4. After evaporation of solvents, the crude product was purified with flash column chromatography on silica gel 60 in a gradient of MeOH in DCM (0 to 3%). ^1H NMR (700 MHz, $CDCl_3$): 7.61 (d, 2H), 7.27 (bs, NH), 7.23 (d, 2H), 2.20 (s, 3H).

To a triflate derivative of acetaminophen (450 mg, 1.59 mM) in anhydrous dioxane (3 cm^3) bis(pinacolato)diboron (564 mg, 2.22 mM, 1.4 eq) was added under argon. After 10 min of argon purging, [1,1′-Bis(diphenylphosphino)ferrocene]dichloropalladium(II) complex with dichloromethane (130 mg, 0.16 mM, 0.1 eq) followed by potassium acetate (468 mg, 4.77 mM, 3 eq) were added and the reaction mixture was refluxed overnight under ambient atmosphere. Next, the solution was cooled down to room temperature and filtered through a short Celite plug. Finally, the plug was washed with a DCM/EtOAc (1:1 v/v) mixture, solvents were evaporated under reduced pressure, and the crude mixture was subjected to flash column chromatography (silica gel 60; MeOH in DCM gradient from 0 to 3%). ^1H NMR (700 MHz, $CDCl_3$): 7.77 (d, 2H), 7.51 (d, 2H), 7.20 (bs, NH), 2.18 (s, 3H), 1.33 (s, 12H).

To obtain the boronic acid form (AAPBA), the pinacolatoboronic derivative (490 mg, 1.77 mM) was transferred to a round-bottom flask containing ammonium acetate (694 mg, 9 mM, 5 eq) and sodium periodate (1.925 g, 9 mM, 5 eq) with acetone (24 cm^3). Water (12 cm^3) was added, and the mixture was allowed to sit overnight. TLC (95:5 DCM:MeOH, v/v) showed completion of the reaction. Acetone was evaporated and the aqueous solution was extracted with EtOAc. After drying with sodium sulfate, solvents were removed, and the crude mixture was purified by flash column chromatography (silica gel 60; MeOH in DCM gradient from 0 to 5%). ^1H NMR (700 MHz, DMSO-d_6): 8.76 (s, NH), 7.68 (d, 2H), 7.53 (d, 2H), 3.60 (s, 2H), 2.04 (s, 3H). MS ESI: calc. for $C_8H_{11}BNO_3$ $[M + H]^+$ 180.0827, found 180.0789.

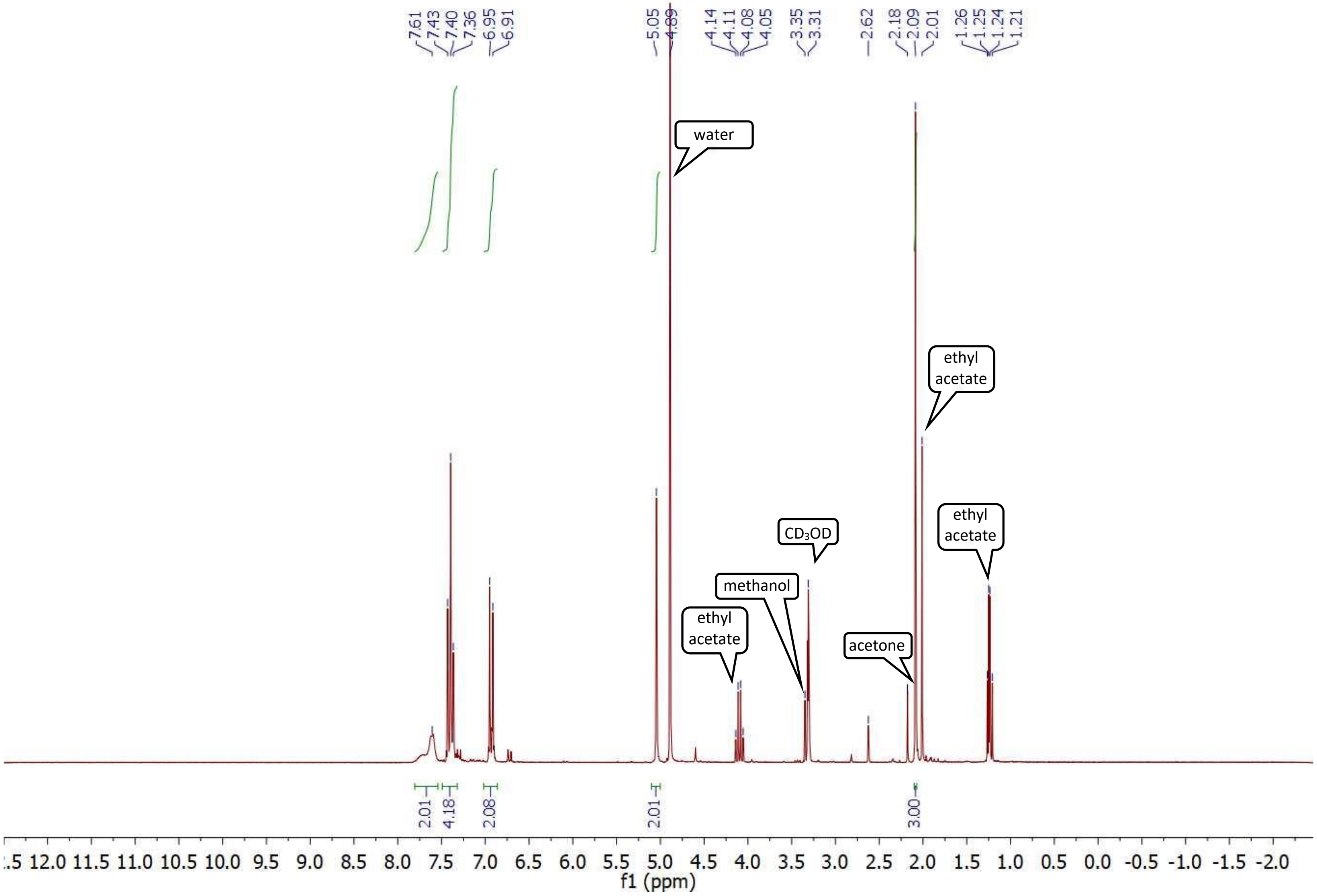

Supplementary Figure S1. ^{1}H NMR of AMBB.

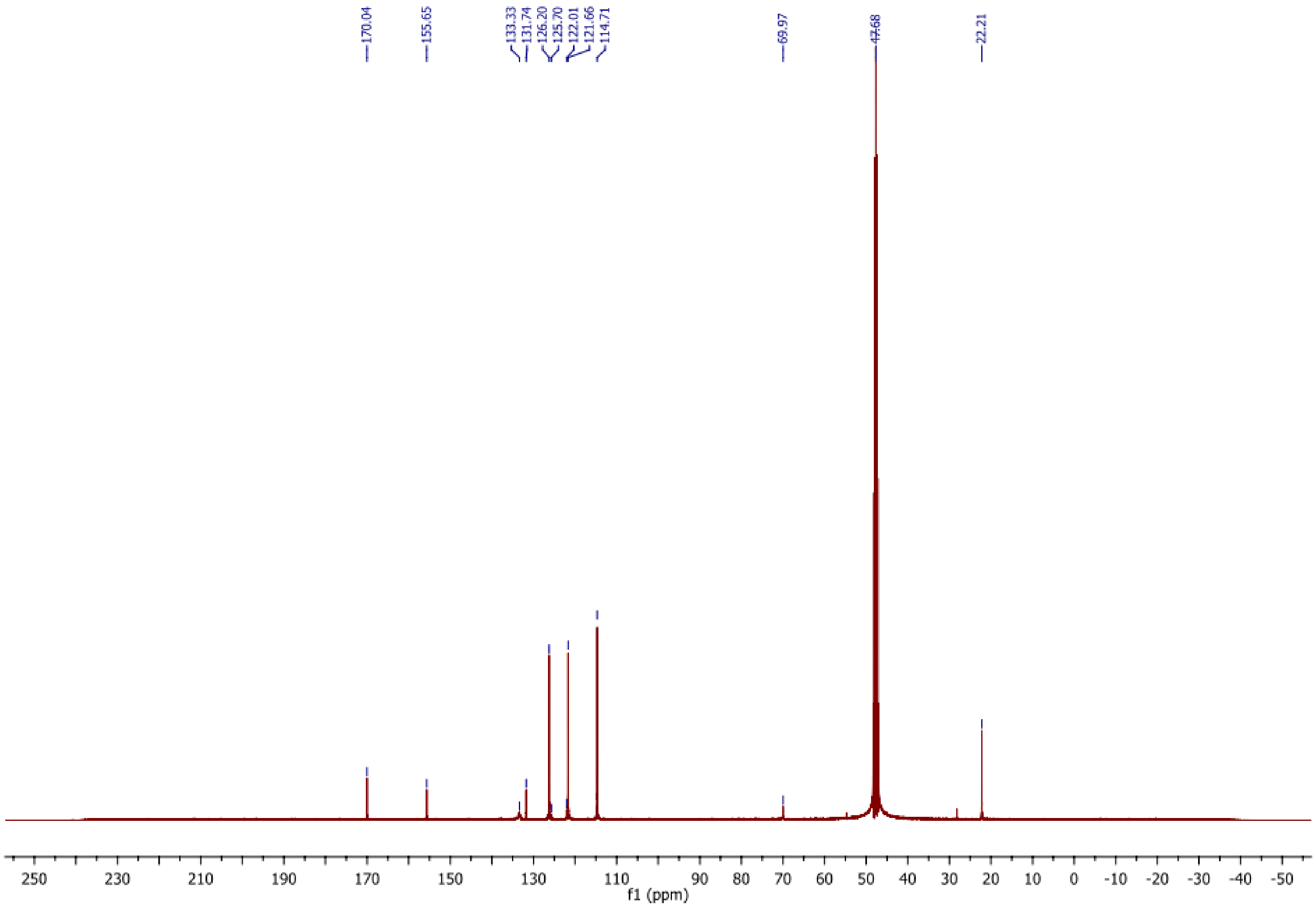

Supplementary Figure S2. ^{13}C NMR of AMBB.

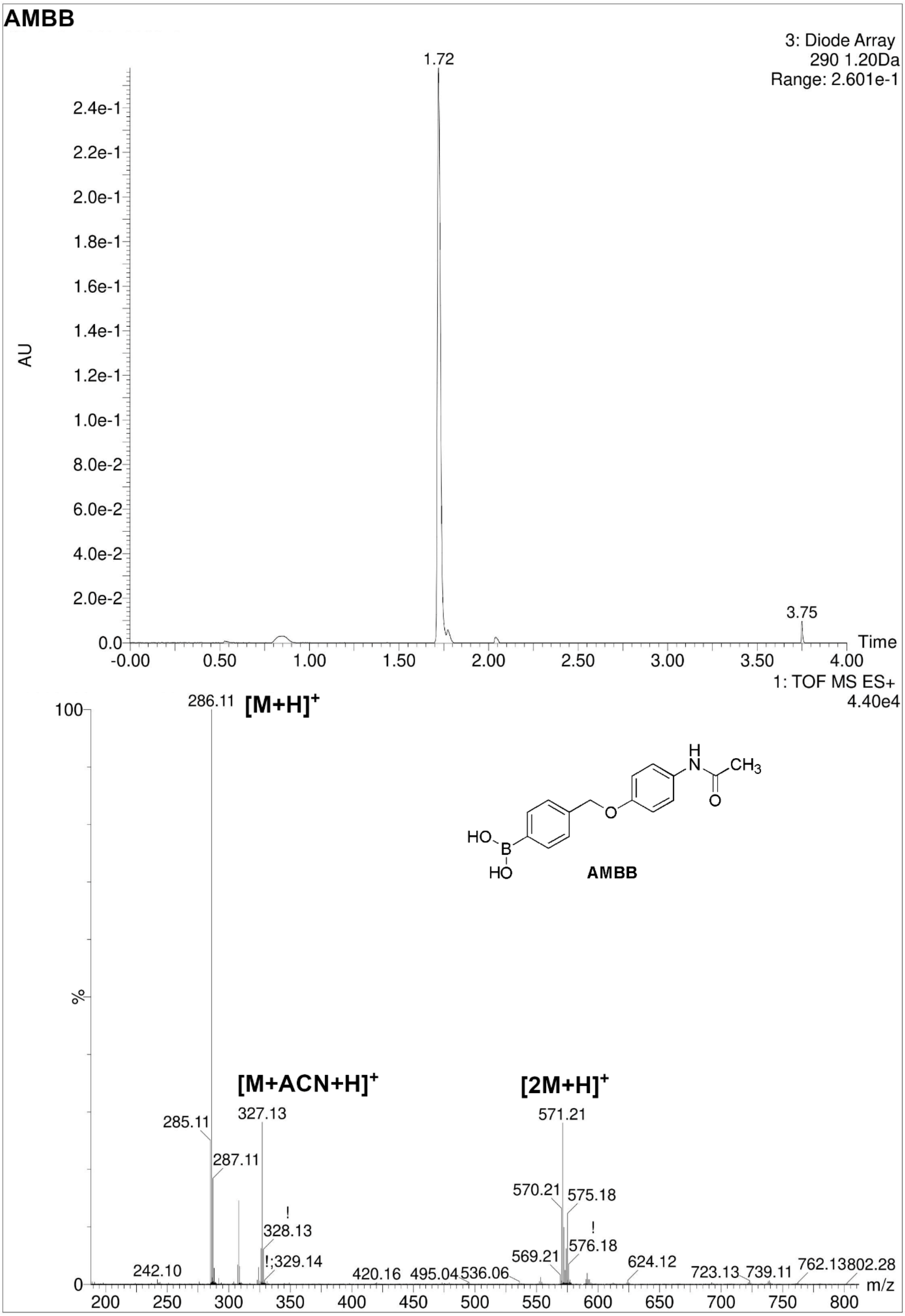

Supplementary Figure S3. UPLC purity trace for AMBB (upper panel) and ESI(+) mass spectrum of AMBB (lower panel).

Elemental Composition Report

Single Mass Analysis
Tolerance = 2.0 mDa / DBE: min = -0.5, max = 50.0
Element prediction: Off
Number of isotope peaks used for i-FIT = 4

Monoisotopic Mass, Even Electron Ions
1394 formula(e) evaluated with 10 results within limits (up to 50 best isotopic matches for each mass)
Elements Used:
C: 0-500 H: 0-1000 B: 0-2 N: 0-200 O: 0-200

JP_2_37_02072018_7 274 (1.807)

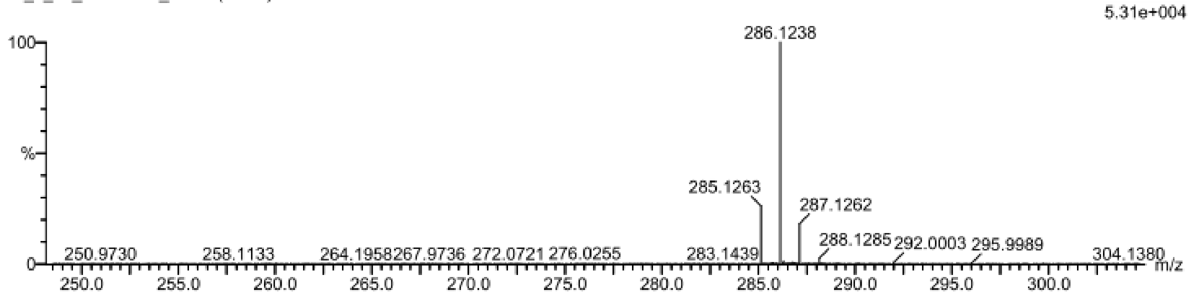

Mass	Calc. Mass	mDa	PPM	DBE	i-FIT	i-FIT (Norm)	Formula
Minimum:				-0.5			
Maximum:		2.0	10.0	50.0			
286.1238	286.1251	-1.3	-4.5	8.5	495.2	0.0	C15 H17 B N O4
	286.1224	1.4	4.9	9.5	498.9	3.7	C11 H13 B N7 O2
	286.1232	0.6	2.1	13.5	500.7	5.5	C20 H16 N O
	286.1250	-1.2	-4.2	0.5	503.0	7.8	C8 H20 N3 O8
	286.1237	0.1	0.3	6.5	503.7	8.5	C5 H12 N13 O2
	286.1224	1.4	4.9	1.5	504.6	9.4	C4 H16 N9 O6
	286.1256	-1.8	-6.3	1.5	510.4	15.3	H13 B N13 O5
	286.1243	-0.5	-1.7	4.5	513.7	18.5	C6 H14 B2 N7 O5
	286.1229	0.9	3.1	-0.5	514.7	19.5	C5 H18 B2 N3 O9
	286.1256	-1.8	-6.3	9.5	515.5	20.3	C7 H10 B2 N11 O

Supplementary Figure S4. High-resolution mass spectrum for AMBB with elemental composition report for AMBB.

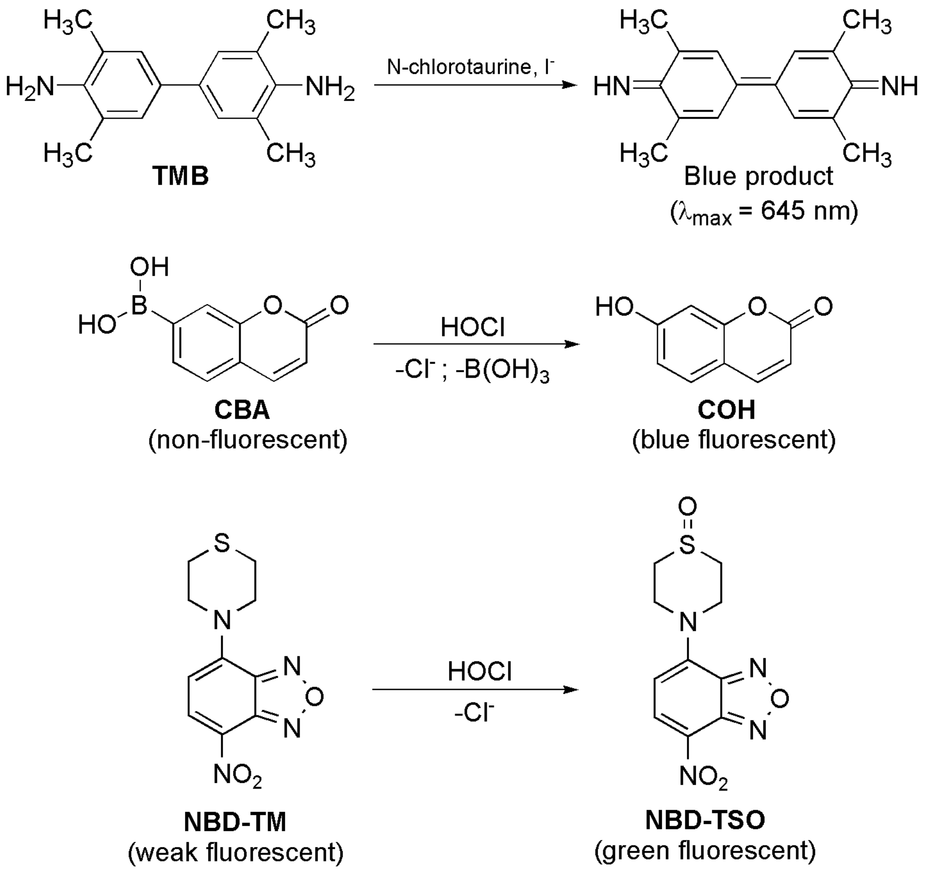

Supplementary Figure S5. Chemical structures of TMB, TMB oxidation product, CBA, COH, NBD-TM, and NBD-TSO.

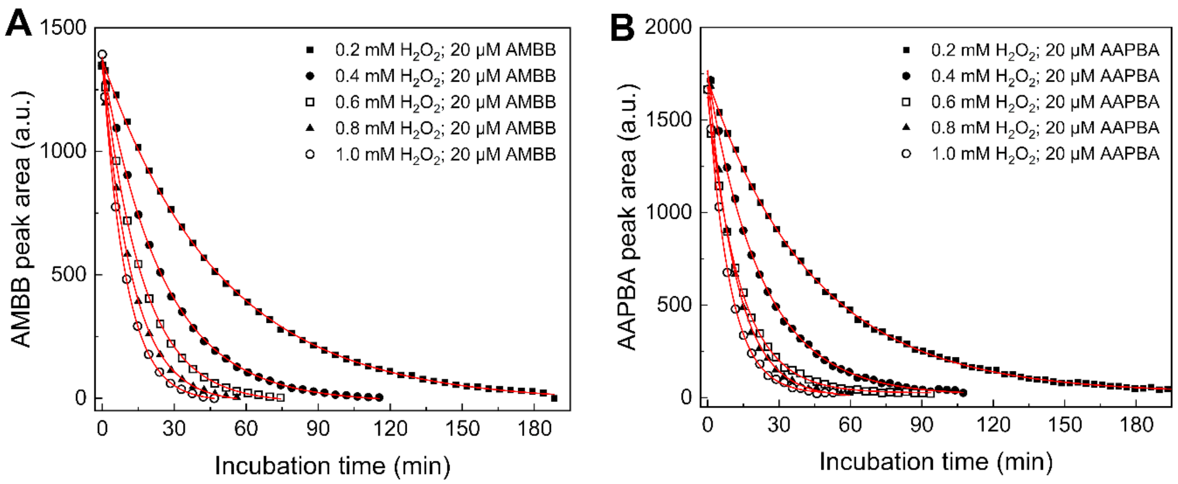

Supplementary Figure S6. Kinetic traces of AMBB (**A**) and AAPBA (**B**) decay measured by UPLC along with fitted exponential curves according to a pseudo-first order equation. Incubation mixtures contained 20 µM AMBB or AAPBA, 0.2–1 mM H_2O_2, 20 mM phosphate buffer (pH 7.4), and 2.5% (v/v) CH_3CN.

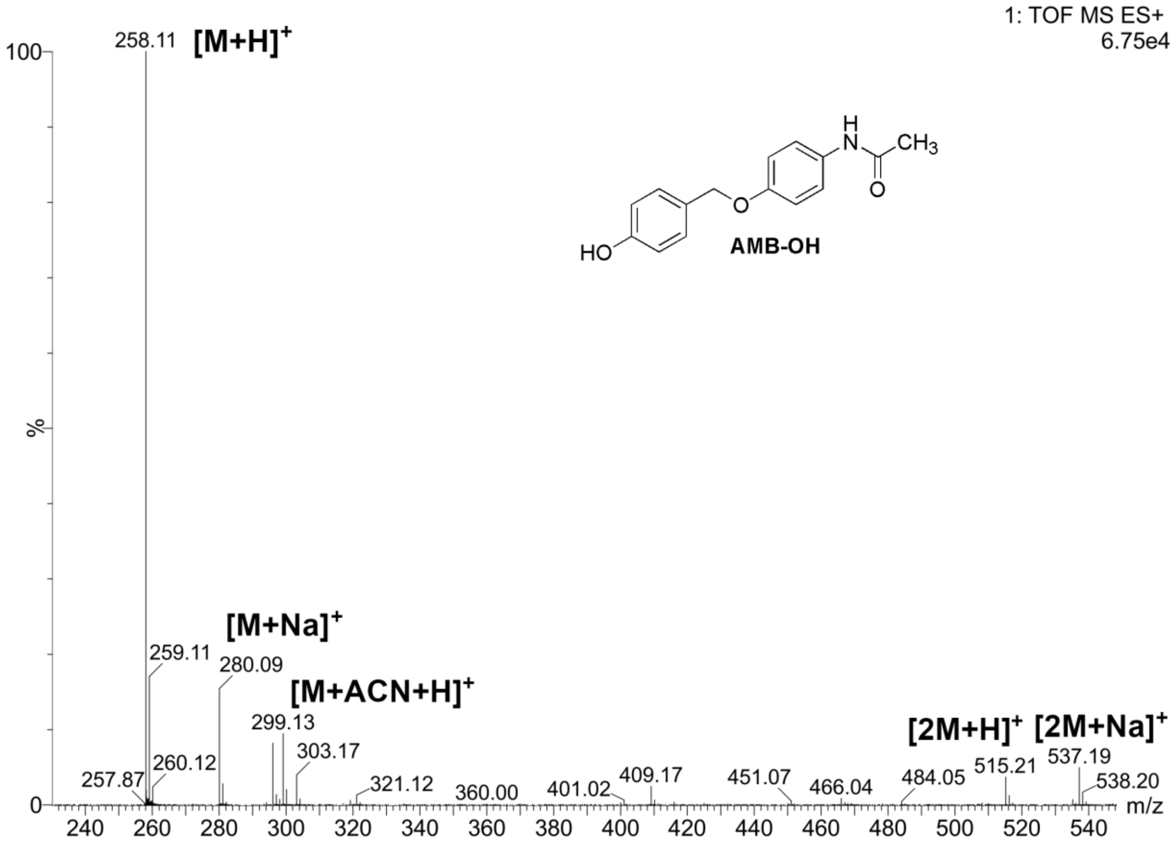

Elemental Composition Report

Page 1

Single Mass Analysis

Tolerance = 6.0 mDa / DBE: min = -1.5, max = 50.0
Element prediction: Off
Number of isotope peaks used for i-FIT = 3

Monoisotopic Mass, Even Electron Ions
401 formula(e) evaluated with 9 results within limits (up to 50 best isotopic matches for each mass)
Elements Used:
C: 0-500 H: 0-1000 N: 0-200 O: 0-200
20mM P.B.; 2.5% ACN;50µM AMBB;55µM ONOO-
AMBB_ONOO_24022023_30 307 (2.043) Cm (304:315-(318:333+292:301))

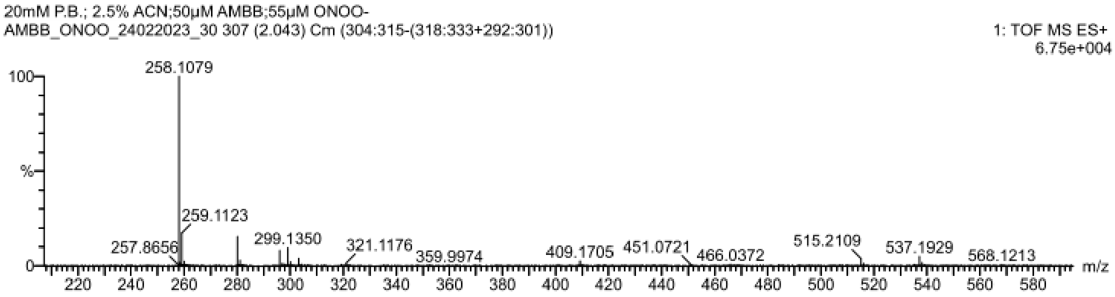

Minimum:				-1.5			
Maximum:		6.0	10.0	50.0			
Mass	Calc. Mass	mDa	PPM	DBE	i-FIT	i-FIT (Norm)	Formula
258.1079	258.1130	-5.1	-19.8	8.5	514.9	0.0	C15 H16 N O3
	258.1031	4.8	18.6	13.5	519.8	4.9	C17 H12 N3
	258.1103	-2.4	-9.3	9.5	521.6	6.7	C11 H12 N7 O
	258.1090	-1.1	-4.3	4.5	522.1	7.2	C10 H16 N3 O5
	258.1063	1.6	6.2	5.5	525.8	10.8	C6 H12 N9 O3
	258.1050	2.9	11.2	0.5	526.0	11.1	C5 H16 N5 O7
	258.1023	5.6	21.7	1.5	529.2	14.2	C H12 N11 O5
	258.1036	4.3	16.7	6.5	529.2	14.3	C2 H8 N15 O
	258.1135	-5.6	-21.7	1.5	530.0	15.1	H12 N13 O4

Supplementary Figure S7. ESI(+) mass spectrum of AMB-OH (upper panel) and elemental composition report for AMB-OH (lower panel).

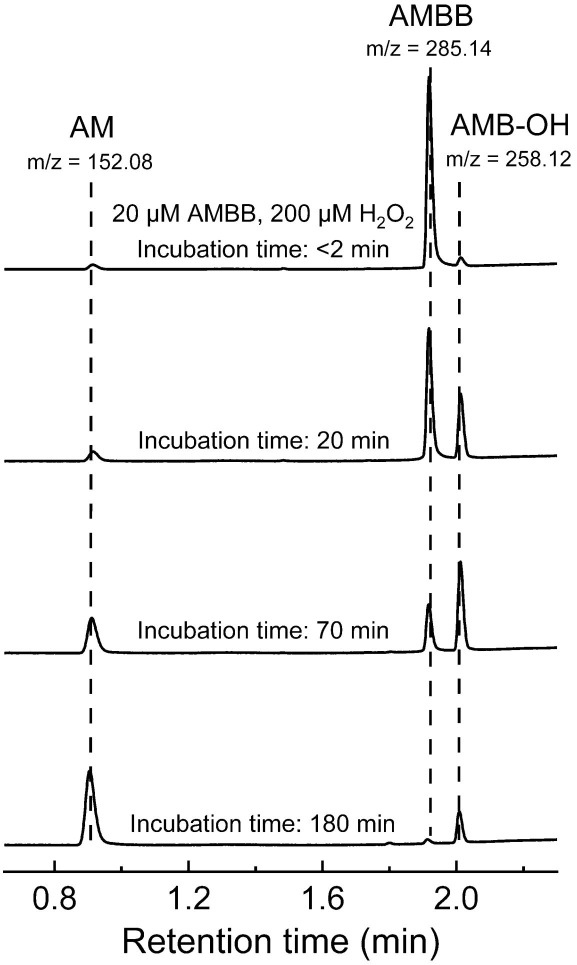

Supplementary Figure S8. UPLC traces of a sample containing 20 µM AMBB, 200 µM H_2O_2, 20 mM phosphate buffer (pH 7.4), and 2.5% (v/v) CH_3CN. Chromatograms extracted at 250 ± 1.2 nm.

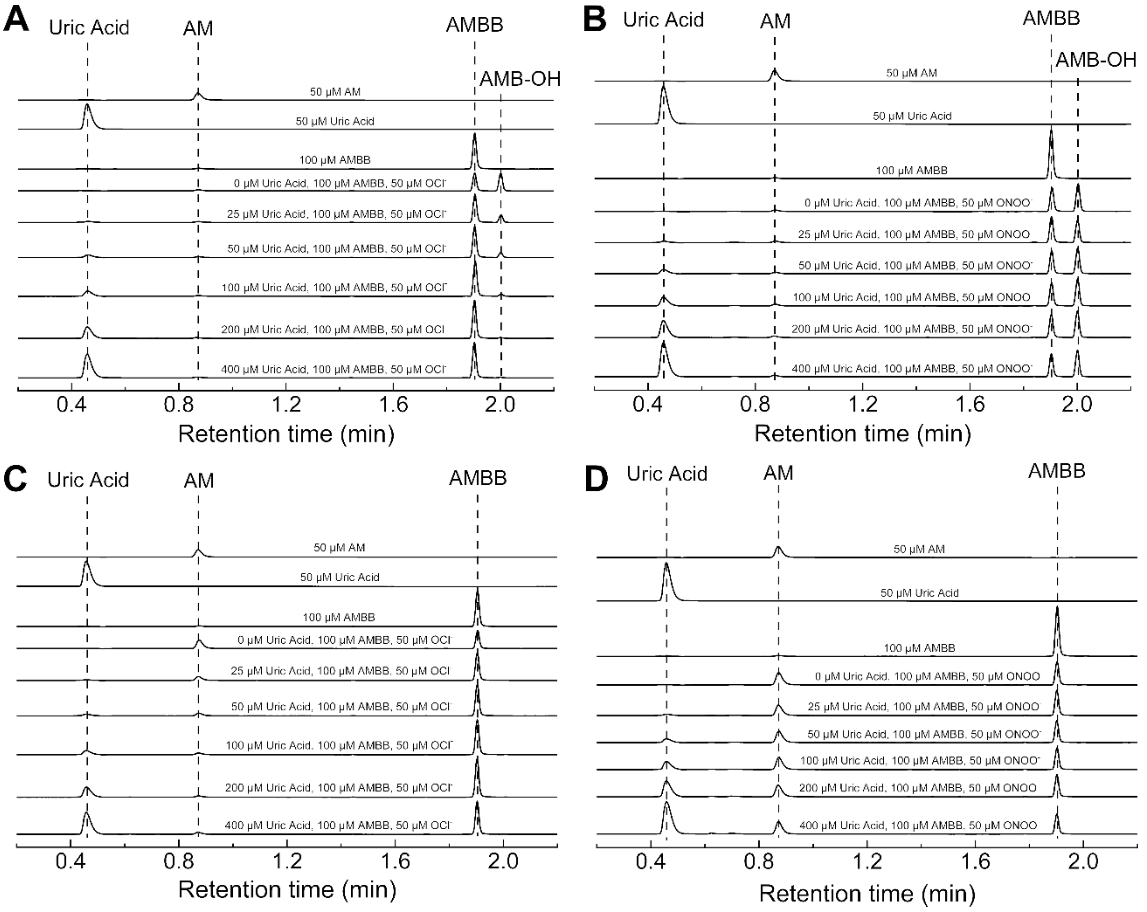

Supplementary Figure S9. Effect of uric acid on the oxidation of AMBB by HOCl and $ONOO^-$. (**A**) UPLC traces for the mixtures containing uric acid (0 – 400 µM), AMBB (100 µM), HOCl (50 µM), phosphate buffer (20 mM, pH 7.4), and 5% (v/v) CH_3CN. Samples analyzed immediately (<2 min) after mixing. (**B**) same as (**A**) but $ONOO^-$ (50 µM) was added instead of HOCl. (**C**) same as (**A**) but samples analyzed 24 h after mixing. (**D**) same as (**B**) but samples analyzed 24 h after mixing.

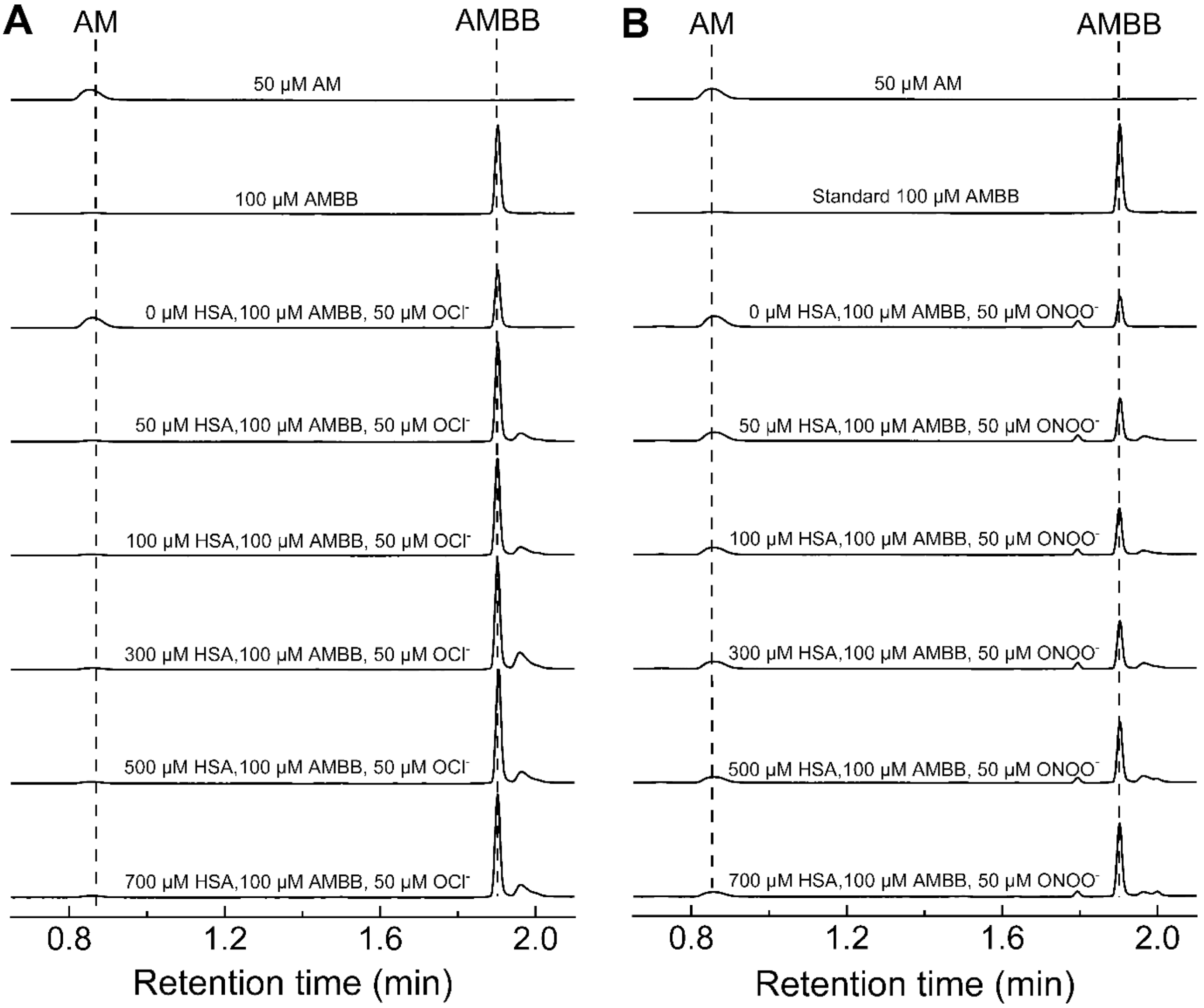

Supplementary Figure S10. Effect of HSA on the oxidation of AMBB by HOCl and $ONOO^-$. (**A**) UPLC traces for the mixtures containing HSA (0–700 μM), AMBB (100 μM), HOCl (50 μM), phosphate buffer (20 mM, pH 7.4), and 5% (v/v) CH_3CN. Samples analyzed 24 h after mixing. (**B**) same as (**A**) but $ONOO^-$ (50 μM) was added instead of HOCl.

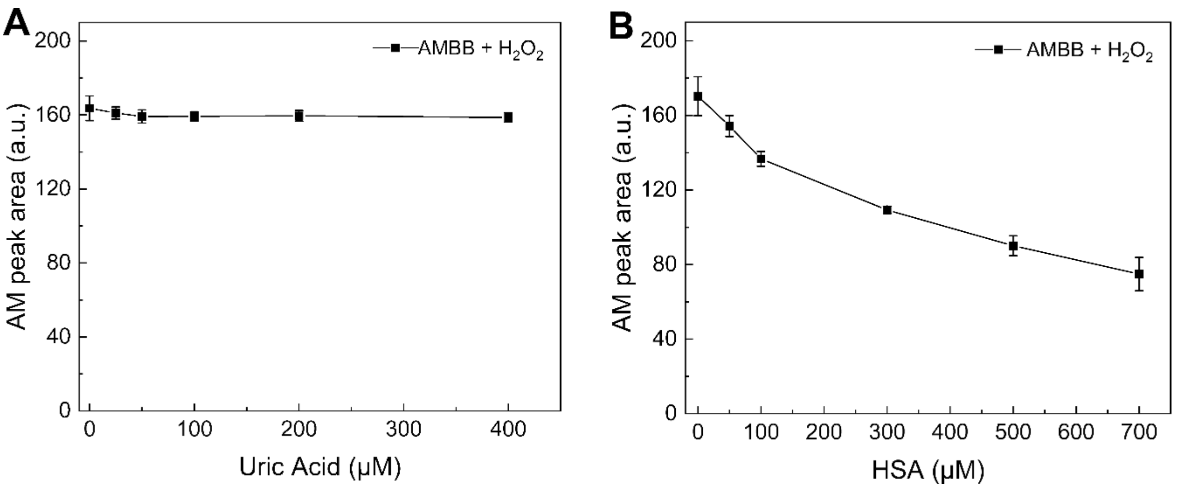

Supplementary Figure S11. Effect of uric acid and human serum albumin (HSA) on the extent of oxidation of AMBB by H_2O_2. (**A**) UPLC peak areas of acetaminophen (AM). Incubation mixtures contained 100 μM AMBB, 50 μM H_2O_2, 0–400 μM uric acid, 20 mM phosphate buffer (pH 7.4), and 5% (v/v) CH_3CN. Samples were analyzed 8 h after mixing. (**B**) UPLC peak areas of AM. Incubation mixtures contained 100 μM AMBB, 50 μM H_2O_2, 0–700 μM HSA, 20 mM phosphate buffer (pH 7.4), and 5% (v/v) CH_3CN. Samples were analyzed 24 h after mixing. UPLC peak areas were integrated for chromatograms extracted at 250 ± 5 nm. Points represent means ± S.D. for three independent measurements.

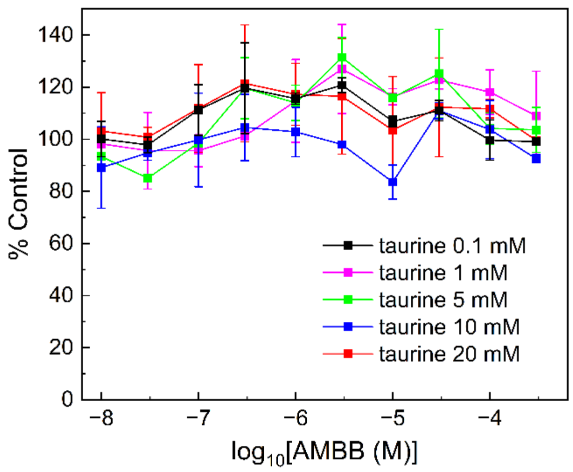

Supplementary Figure S12. Dose-response curves for AMBB determined by the taurine N-chloramine/TMB assay. Mixtures contained taurine (0.1–20 mM), MPO (0.1 nM, 5 nM HOCl/s), hydrogen peroxide (10 μM), NaCl (0.1 M), phosphate buffer (20 mM, pH 7.4), 3% (v/v) MeOH, and AMBB (0.01 μM–300 μM). The experimental points were read for the incubation time of 5 min.
